# Benzophenones-natural metabolites with great Hopes in drug discovery: structures, occurrence, bioactivities, and biosynthesis

**DOI:** 10.1039/d3ra02788k

**Published:** 2023-08-04

**Authors:** Sabrin R. M. Ibrahim, Duaa Fahad ALsiyud, Abdulrahman Y. Alfaeq, Shaimaa G. A. Mohamed, Gamal A. Mohamed

**Affiliations:** a Preparatory Year Program, Department of Chemistry, Batterjee Medical College Jeddah 21442 Saudi Arabia sabrin.ibrahim@bmc.edu.sa +966-581183034; b Department of Pharmacognosy, Faculty of Pharmacy, Assiut University Assiut 71526 Egypt sabreen.ibrahim@pharm.aun.edu.eg; c Department of Medical Laboratories – Hematology, King Fahd Armed Forces Hospital Corniche Road, Andalus Jeddah 23311 Saudi Arabia duaaalsiyud@yahoo.com; d Pharmaceutical Care Department, Ministry of National Guard – Health Affairs Jeddah 22384 Saudi Arabia Faegab@mngha.med.sa; e Faculty of Dentistry, British University, El Sherouk City Suez Desert Road Cairo 11837 Egypt shaimaag1973@gmail.com; f Department of Natural Products and Alternative Medicine, Faculty of Pharmacy, King Abdulaziz University Jeddah 21589 Saudi Arabia gahussein@kau.edu.sa

## Abstract

Fungi have protruded with enormous development in the repository of drug discovery, making them some of the most attractive sources for the synthesis of bio-significant and structural novel metabolites. Benzophenones are structurally unique metabolites with phenol/carbonyl/phenol frameworks, that are separated from microbial and plant sources. They have drawn considerable interest from researchers due to their versatile building blocks and diversified bio-activities. The current work aimed to highlight the reported data on fungal benzophenones, including their structures, occurrence, and bioactivities in the period from 1963 to April 2023. Overall, 147 benzophenones derived from fungal source were listed in this work. Structure activity relationships of the benzophenones derivatives have been discussed. Also, in this review, a brief insight into their biosynthetic routes was presented. This work could shed light on the future research of benzophenones.

## Abstract 2

Fungi have protruded with enormous development in the repository of drug discovery, making them some of the most attractive sources for the synthesis of bio-significant and structural novel metabolites.

## Graphical abstract

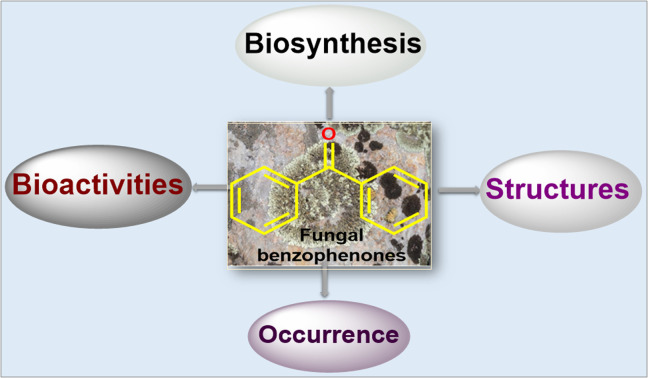

## 1 Introduction

**Fig. 1:**
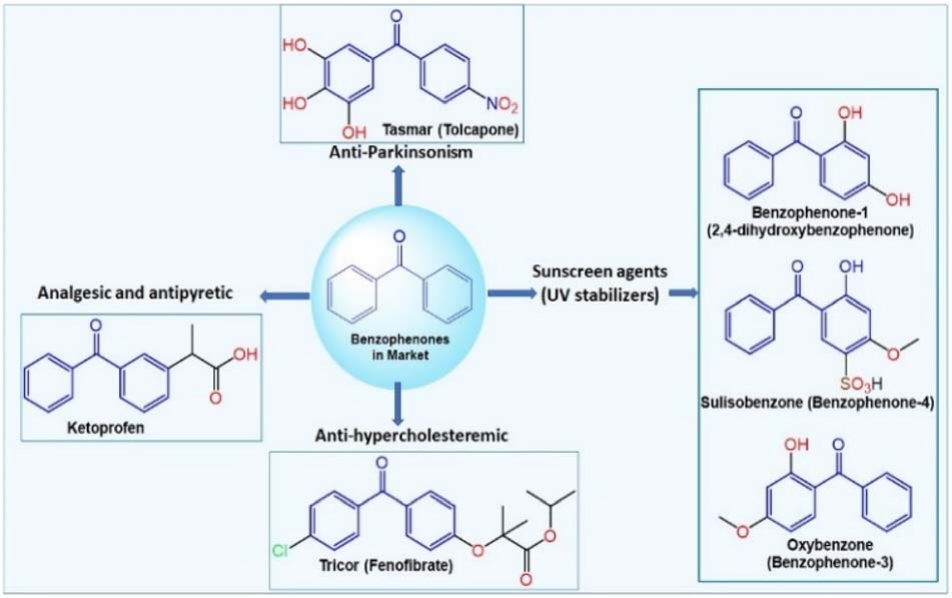
Examples of benzophenone derivatives in the market and their uses.

Fungi are some of the most fundamental and optimistic sources of bio-metabolites, apparently due to the biodiversity and chemical divergence of their metabolites that could be employed for pharmacological applications and drug discovery.^1–4^ Yet, a huge number of metabolites with unique structural skeletons and prominent effectiveness have been found in fungi, making them one of the fascinating repositories for therapeutics and lead scaffolds.^2,5–9^ These metabolites play crucial functions in treating various disorders, such as hypercholesterolemia (statins), autoimmune diseases, cancer, depression, and infections (antibiotics and antifungal medications).^5–9^ Benzophenones (BPs) are a wide class of natural metabolites that have been reported from fungi or higher plants of different families (*e.g.*, Clusiaceae, Iridaceae, Lauraceae, Rosaceae, Moraceae, Daphneceae, and Myrtle families).^10–13^ They have phenol/carbonyl/phenol frameworks that are commonly involved in the skeletons of various natural metabolites. Many of the reported derivatives are either polyprenylated or dimeric benzophenone derivatives. Natural BPs without side chains are of rare occurrence. These metabolites are linked with OMe, –OH, prenyl, or geranyl groups. Interestingly, these metabolites possess an active carbonyl, thus they can easily react with other functionalities to form a variety of new skeletons.^14^ Recently, new polyprenylated BPs with unusual, rearranged skeletons were reported from certain fungi and higher plants.^10^ The research on these metabolites attracts remarkable attention due to their structural variety and diverse bio-activities such as protein kinase, sterol *O*-acyltransferase, α-glucosidase, proteasome, and tyrosine phosphatase inhibitory activity, plant growth inhibition, anti-nematode, antimicrobial, anti-mycobacterial, antialgal, anticoccidial, cytotoxic, anti-malarial, phytotoxic, antioxidant, anti-inflammation, anti-osteoclastogenic, antihyperlipidemic, immune-suppressive, and insecticidal. Additionally, they have a rich nucleophilic nucleus that could inspire many chemists and pharmacologists to synthesize more related derivatives and generate a novel compound library for developing new medicines to treat various health-related disorders.^15^ In 2018, Surana *et al.* reviewed the reported synthetic strategies for benzophenone and its derivatives.^15^ Due to their better UV protection capacity, FDA (US Food and Drug Administration) and some countries have approved their use as ingredients in sunscreen combinations.^13^ Also, BPs are widely included in personal care preparations (*i.e.*, shampoos, toothpaste, sanitation products, body washes, makeup, and skin lotion) to keep the colour and scents of these preparations, as well as UV light absorbers in synthetic products such as paints and insecticides, which are exposed to sunlight.^11,13^ Interestingly, some BPs derivatives are available as commercial drugs such as tolcapone (Tasmar, anti-Parkinson`s disease), ketoprofen (analgesic and antipyretic), fenofibrate (Tricor, anti-hypercholesteraemic), and sulisobenzone, benzophenone-1 (BP-1, 2,4-dihydroxybenzophenone), and oxybenzone (benzophenone-3, 2-hydroxy-4-methoxybenzophenone) (sunscreen agents)^15^ (Fig. 1). Commonly, BP-3 and BP-1 are utilized as stabilizers to prevent photodegradation in many commercial products and as UV filters in cosmetics and sunscreens to prohibit skin damage and sunburn.^16^

Various reviews focused on BPs reported from various plant families particularly those from family Clusiaceae, including their chemistry, structural determination, and bioactivities.^10,12,17^ Also, in 2019, Mao *et al.* summarized the reported studies regarding the BPs's occurrence and fate in the aquatic systems.^13^ It was noted that there no comprehensive review covering BPs reported from fungal origin. Therefore, the current work focused on the BPs reported from various fungal species, including their structures, sources, host, occurrence, biosynthesis, and bioactivities in the period from 1963 to April 2023 (Table 1). Here, we intended to introduce together all current knowledge on fungal benzophenones aiming at understanding and rationalizing their bioactivities, structures, and biosynthesis for their possible usage as leads for the synthesis and development of pharmaceutical agents.

**Table 1:** Naturally occurring fungal benzophenones (fungal source, host, place, molecular weights, and formulae)^a^

Compound name	Fungus	Host (part)	Source, place	Ref.
Moniliphenone (1)	*Monilinia fructicola*	—	Cultured	18
	*Hypocreales* (MSX 17022)	Leaf litter from a beech tree community	Hillsborough, NC, USA	19
	*Penicillium citrinum* (PSU-RSPG95)	Soil sample	Rajjaprabha Dam, Surat Thani, Thailand	20
	*Fimetariella rabenhorstii* (SR84-1C)	*Quercus brantii* (stems, Fagaceae)	Natural area in Kurdistan, Iran	21
	*Alternaria sonchi* (S-102)	*Sonchus arvensis* (leaves, Asteraceae)	Russia	22
Rabenzophenone (2) = 5-chloromoniliphenone	*Fimetariella rabenhorstii* (SR84-1C)	*Quercus brantii* (stems, Fagaceae)	Natural area in Kurdistan, Iran	21
	*Alternaria sonchi* (S-102)	*Sonchus arvensis* (leaves, Asteraceae)	Russia	22
4-Hydroxy-2-(2-hydroxy-3-methoxy-5-methylbenzoyl)-6-methoxybenzaldehyde (3)	*Daldinia concentrica*	—	Tokushima	23
2-(2,3-Dimethoxy-5-methylbenzoyl)-4-hydroxy-6-methoxybenzaldehyde (4)	*Daldinia concentrica*	—	Tokushima	23
Nidulalin B (5)	*Emericella nidulans* var. *lata* (IN 68) = *Aspergillus nidulellus*	*Trigonella foenumgraecum* (Fabaceae)	Indonesia	24
Cercophorin A (6)	*Cercophora areolata* (JS 166 = UAMH 7495)	Porcupine dung	Near Bird Lake, Muskoka District, Ontario, Canada	25
Pestalaphenone A (7)	*Pestalotiopsis* sp.	*Melia azedarach* (stem bark, Meliaceae)	Nanjing, Jiangsu, China	26
Sulochrin (8)	*Aspergillus* sp.	Leaf litter	Near Perth, Western Australia	27
	*Aureobasidium* sp.	Litter layer	Hirosawa, Japan	28
	*Penicillium* sp. (PSU-RSPG99)	Soil sample	Rajjaprabha Dam, Surat Thani, Thailand	29
	*Aspergillus europaeus* (WZXY-SX-4-1)	*Xestospongia testudinaria* (sponge, Petrosiidae)	Weizhou Island, Guangxi, China	30
	*Penicillium citrinum* (HL-5126)	*Bruguiera sexangula* var. *rhynchopetala* (Mangrove plant, Rhizophoraceae)	South China Sea	31
	*Penicillium* sp.	*Acanthus ilicifolius* (Mangrove plant, Acanthaceae)	Beibu gulf, Guangxi, China	32
	*Aspergillus fumigatus* (GZWMJZ-152)	Piece of 35 m-deep cave soil	Fanjing, Mountain of Guizhou, China	33
	*Aspergillus flavipes* (PJ03-11)	Wetland mud	Panjin Red Beach National Nature Reserve, Liaoning, China	34
Demethylsulochrin (9)	*Aspergillus* sp.	Leaf litter	Near Perth, Western Australia	27
Monomethylsulochrin (10)	*Rhizoctonia* sp. (Cy064)	*Cynodon dactylon* (leaves, Poaceae)	Jiangsu, China	35
	*Guignardia* sp. (IFB-E028)	*Hopea hainanensis* (leaves, Dipterocarpaceae)	Hainan Island, China	36
	*Aspergillus fumigatus*	*Solanum insanum* (fruit, Solanaceae)	Central Province of Sri Lanka	37
	*Aspergillus fumigatus* (GZWMJZ-152)	Piece of 35 m-deep cave soil	Fanjing, Mountain of Guizhou, China	33
3,5-Dichlorosulochrin (11)	*Aspergillus flavipes* (PJ03-11)	Wetland mud	Panjin Red Beach National Nature Reserve in Liaoning, China	38
3-de-*O*-Methylsulochrin (12)	*Aspergillus flavipes* (PJ03-11)	Wetland mud	Panjin Red Beach National Nature Reserve in Liaoning, China	38
	*Aspergillus europaeus* (WZXY-SX-4-1)	*Xestospongia testudinaria* (sponge, Petrosiidae)	Weizhou Island, Guangxi, China	30
14-de-*O*-Methyl-5-methoxysulochrin (13)	*Aspergillus europaeus* (WZXY-SX-4-1)	*Xestospongia testudinaria* (sponge, Petrosiidae)	Weizhou Island, Guangxi, China	30
5-Methoxysulochrin (14)	*Aspergillus europaeus* (WZXY-SX-4-1)	*Xestospongia testudinaria* (sponge, Petrosiidae)	Weizhou Island, Guangxi, China	30
14-*O*-Demethylsulochrin (15)	*Aspergillus europaeus* (WZXY-SX-4-1)	*Xestospongia testudinaria* (sponge, Petrosiidae)	Weizhou Island, Guangxi, China	30
Hydroxysulochrin (16)	*Aureobasidium* sp.	Litter layer	Hirosawa, Japan	28
	*Penicillium* sp.	*Acanthus ilicifolius* (Mangrove plant, Acanthaceae)	Beibu gulf, Guangxi, China	32
Penibenzophenone A (17)	*Penicillium citrinum* (HL-5126)	*Bruguiera sexangula* var. *rhynchopetala* (Mangrove plant, Rhizophoraceae)	South China Sea	31
Penibenzophenone B (18)	*Penicillium citrinum* (HL-5126)	*Bruguiera sexangula* var. *rhynchopetala* (plant, Rhizophoraceae)	The South China Sea	31
Penibenzophenone C (19)	*Penicillium* sp.	*Acanthus ilicifolius* (plant, Acanthaceae)	Beibu gulf, Guangxi, China	32
Penibenzophenone D (20)	*Penicillium* sp.	*Acanthus ilicifolius* (plant, Acanthaceae)	Beibu gulf, Guangxi, China	32
2-(3,5-Dichloro-2,6-dihydroxy-4-methylbenzoyl)-5-hydroxy-3-methoxybenzoic acid (21)	*Aspergillus flavipes* (PJ03-11)	Wetland mud	Panjin Red Beach National Nature Reserve in Liaoning, China	38
2-(3-Chloro-4-methyl-γ-resorcyloyl)-5-hydroxy-*m*-anisic acid methyl ester (22) = Monochlorsulochrin	*Penicillium* sp. (PSU-RSPG99)	Soil sample	Rajjaprabha Dam, Surat Thani, Thailand	29
	*Aspergillus flavipes* (DL-11)	Coastal sediment	Dalian, Liaoning, China	39
	*Aspergillus flavipes* (PJ03-11)	Wetland mud	Panjin Red Beach National Nature Reserve, Liaoning, China	34
Dihydrogeodin (23)	*Aspergillus* sp. (F1)	*Trewia nudiflora* (seeds, Euphorbiaceae)	Yunnan, China	40
	*Penicillium* sp. (PSU-RSPG99)	Soil sample	Rajjaprabha Dam, Surat Thani, Thailand	29
	*Penicillium citrinum* (PSU-RSPG95)	Soil sample	Rajjaprabha Dam, Surat Thani, Thailand	20
	*Aspergillus flavipes* (DL-11)	Coastal sediment	Dalian, Liaoning, China	39
	*Aspergillus flavipes* (PJ03-11)	Wetland mud	Panjin Red Beach National Nature Reserve, Liaoning, China	34
Penicillanone (24)	*Penicillium citrinum* (PSU-RSPG95)	Soil sample	Rajjaprabha Dam, Surat Thani, Thailand	20
Rhizoctonic acid (25)	*Rhizoctonia* sp. (Cy064)	*Cynodon dactylon* (leaves, Poaceae)	Jiangsu, China	35
	*Guignardia* sp. (IFB-E028)	*Hopea hainanensis* (leaves, Dipterocarpaceae)	Hainan Island, China	36
Astrophenone (26)	*Astrocystis* sp. (BCC 22166)	Mangrove palm Nypa	Hat Khanom-Mu Ko Thale Tai National Park, Nakhon Si Thammarat, Thailand	41
Monodictyphenone (27)	*Monodictys putredinis* (187/195 15 I)	Marine green alga	Tenerife, Spain	42
	*Penicillium* sp. (MA-37)	*Bruguiera gymnorrhiza* (soil, Rhizophoraceae)	Hainan Island, China	43
	*Penicillium albo-biverticillium* (TPU1432)	Unidentified ascidian	Manado, Indonesia	44
Iso-Monodictyphenone (28)	*Penicillium* sp. (MA-37)	*Bruguiera gymnorrhiza* (soil, Rhizophoraceae)	Hainan Island, China	43
Acremonidin E (29)	*Acremonium* sp. (LL-Cyan 416)	—	—	45
Arugosin F (30)	*Aspergillus nidulans* (FGSC A4)	—	Marburg, Germany	46
Maclurin (31)	*Aspergillus nidulans* (FGSC A4)	—	Marburg, Germany	46
1,5,8-Trihydroxybenzophenone (32)	*Aspergillus nidulans* (FGSC A4)	—	Marburg, Germany	46
5-Hydroxy-1,10-dimethoxy-6-carboxybenzophenone (33)	*Aspergillus nidulans* (FGSC A4)	—	Marburg, Germany	46
5-Hydroxy-1,10-dimethoxy-6-carboxylmethylbenzophenone (34)	*Aspergillus nidulans* (FGSC A4)	—	Marburg, Germany	46
2-(2,6-Dihydroxy-4-methylbenzoyl)-6-hydroxybenzoic acid (35)	*Graphiopsis chlorocephala*	*Paeonia lactiflora* (leaves, Paeoniaceae)	Tohoku University, Japan	47
Cephalanone F (36)	*Graphiopsis chlorocephala*	*Paeonia lactiflora* (leaves, Paeoniaceae)	Tohoku University, Japan	47
2,2′,3,5-Tetrahydroxy-3′-methylbenzophenone (37)	*Talaromyces islandicus* (EN-501)	*Laurencia okamurai* (red alga, Rhodomelaceae)	Coast of Qingdao, China	48
2,2′,5′-Trihydroxy-3-methoxy-3′-methylbenzophenone (38)	*Talaromyces islandicus* (EN-501)	*Laurencia okamurai* (red alga, Rhodomelaceae)	Coast of Qingdao, China	48
Peniphenone (39)	*Penicillium* sp. (ZJ-SY2)	*Sonneratia apetala* (leaves, Lythraceae)	Zhanjiang Mangrove Nature Reserve, Guangdong, China	49
Methyl peniphenone (40)	*Penicillium* sp. (ZJ-SY2)	*Sonneratia apetala* (leaves, Lythraceae)	Zhanjiang Mangrove Nature Reserve, Guangdong, China	49
Methyl 2-(2,6-dihydroxy-4-methylbenzoyl)-3-hydroxy-5-methoxybenzoate (41)	*Ascomycota* sp. (SK2YWS-L)	*Kandelia cande* (leaf, Rhizophoraceae)	Shankou Mangrove Nature Reserve, Guangxi, China	50
Preacredinone A (42)	*Acremonium* sp. (F9A015)	*Suberites japonicus* (sponge, Suberitidae)	Ga-geo Island near the southwest sea of Korea	51
Cytosporaphenone A (43)	*Cytospora rhizophorae* (A761)	*Morinda officinalis* (twigs, Rubiaceae)	Gaoyao, Guangdong, China	52
Orbiophenone A (44)	*Orbiocrella petchii* (BCC 51377)	A scale-insect (Hemiptera) underside of a leaf (Poaceae)	Chae Son National Park, Lampang, Thailand	53
Cytorhizophin C (45)	*Cytospora rhizophorae* (A761)	*Morinda officinalis* (twigs, Rubiaceae)	Gaoyao, Guangdong, China	54
	*Fimetariella rabenhorstii* (SR84-1C)	*Quercus brantii* (stems, Fagaceae)	Natural area in Kurdistan (Iran)	21
Rhizophol A (46)	*Cytospora rhizophorae* (A761)	*Morinda officinalis* (twigs, Rubiaceae)	Gaoyao, Guangdong, China	55
Eurobenzophenone A (47)	*Aspergillus europaeus* (WZXY-SX-4-1)	*Xestospongia testudinaria* (sponge, Petrosiidae)	Weizhou Island, Guangxi, China	30
Eurobenzophenone B (48)	*Aspergillus europaeus* (WZXY-SX-4-1)	*Xestospongia testudinaria* (sponge, Petrosiidae)	Weizhou Island, Guangxi, China	30
Eurobenzophenone C (49)	*Aspergillus europaeus* (WZXY-SX-4-1)	*Xestospongia testudinaria* (sponge, Petrosiidae)	Weizhou Island, Guangxi, China	30
Wentiphenone A (50)	*Aspergillus wentii* (WN-11-8-1, WN-11-8-2, WN-11-5-2)	Sediment of a hypersaline lake	Wadi El Natrun, Egypt	56
Pestalotinone A (51)	*Pestalotiopsis trachicarpicola* (SCJ551)	*Blechnum orientale* (stem, Blechnaceae)	Shatoujiao forestry center, Shenzhen, Guangdong, China	57
2,6′-Dihydroxy-2,4′dimethoxy-8′-methyl-6-methoxy-acyl-ethyl-diphenylmethanone (52)	*Aspergillus fumigatus* (SWZ01)	Sea sediment	Shenzhen, Guangdong, China	58
Shiraone A (53)	*Shiraia* sp. (BYJB-1)	*Selaginella delicatula* (leaves, Selaginellaceae)	Huangsang nature reserve, Shaoyang city, Hunan, China	59
Griseophenone B (54)	*Penicillium* sp. (ct-28)	*Corydlis tomentella* (leaves, Papaveraceae)	Jinfo Mountain, Chongqing, China	60,61
Griseophenone C (55)	*Penicillium* sp. (ct-28)	*Corydlis tomentella* (leaves, Papaveraceae)	Jinfo Mountain, Chongqing, China	60,62
	Pleosporales sp. (YY-4)	*Uncaria rhynchophylla* (plant, Rubiaceae)	Jian, Jiangxi, China	63
Griseophenone I (56)	*Penicillium* sp. (ct-28)	*Corydlis tomentella* (leaves, Papaveraceae)	Jinfo Mountain, Chongqing, China	60,62
Sulfurasperine A (57)	*Aspergillus fumigatus* (GZWMJZ-152)	Piece of 35 m-deep cave soil	Fanjing, Mountain of Guizhou, China	33
(±)-Sulfurasperine B (58)	*Aspergillus fumigatus* (GZWMJZ-152)	Piece of 35 m-deep cave soil	Fanjing, Mountain of Guizhou, China	33
(±)-Sulfurasperine C (59)	*Aspergillus fumigatus* (GZWMJZ-152)	Piece of 35 m-deep cave soil	Fanjing, Mountain of Guizhou, China	33
Sulfurasperine D (60)	*Aspergillus fumigatus* (GZWMJZ-152)	Piece of 35 m-deep cave soil	Fanjing, Mountain of Guizhou, China	33
Pleosporone F (61)	Pleosporales sp. (YY-4)	*Uncaria rhynchophylla* (plant, Rubiaceae)	Jian, Jiangxi, China	63
2,4,6-Trihydroxy-2′,4′-dimethoxy-6′-methylbenzophenone (62)	Pleosporales sp. (YY-4)	*Uncaria rhynchophylla* (plant, Rubiaceae)	Jian, Jiangxi, China	63
Pleosporone D (63)	Pleosporales sp. (YY-4)	*Uncaria rhynchophylla* (plant, Rubiaceae)	Jian, Jiangxi, China	63
Pleosporone E (64)	Pleosporales sp. (YY-4)	*Uncaria rhynchophylla* (plant, Rubiaceae)	Jian, Jiangxi, China	63
Cephalanone A (65)	*Graphiopsis chlorocephala*	*Paeonia lactiflora* (leaves, Paeoniaceae)	Tohoku University, Japan	47
Cephalanone B (66)	*Graphiopsis chlorocephala*	*Paeonia lactiflora* (leaves, Paeoniaceae)	Tohoku University, Japan	47
Cephalanone C (67)	*Graphiopsis chlorocephala*	*Paeonia lactiflora* (leaves, Paeoniaceae)	Tohoku University, Japan	47
SB87-H (8-*O*-demethyl-11-dechloropestalone (68)	*Pestalotiopsis trachicarpicola* (SCJ551)	*Blechnum orientale* (stem, Blechnaceae)	Shatoujiao forestry center, Shenzhen, Guangdong, China	57
Tenellone A (69)	*Diaporthe* sp.	*Aeonium cuneatum* (stems, Crassulaceae)	El Pijaral, Tenerife, Canary Islands, Spain	64
	*Phomopsis lithocarpus* (FS508)	Marine sediment	Indian Ocean	65
Tenellone B (70)	*Diaporthe* sp.	*Aeonium cuneatum* (stems, Crassulaceae)	El Pijaral, Tenerife, Canary Islands, Spain	64
Tenellone C (71)	*Diaporthe* sp. (SYSU-HQ3)	*Excoecaria agallocha* (Mangrove plant, Euphorbiaceae)	Zhuhai, Guangdong, China	14
Tenellone D (72)*	*Diaporthe* sp. (SYSU-HQ3)	*Excoecaria agallocha* (Mangrove plant, Euphorbiaceae)	Zhuhai, Guangdong, China	14
Tenellone D (73)**	*Phomopsis lithocarpus* (FS508)	Marine sediment	Indian Ocean	65
Tenellone E (74)	*Phomopsis lithocarpus* (FS508)	Marine sediment	Indian Ocean	65
Tenellone F (75)	*Phomopsis lithocarpus* (FS508)	Marine sediment	Indian Ocean	65
Tenellone G (76)	*Phomopsis lithocarpus* (FS508)	Marine sediment	Indian Ocean	65
Tenellone H (77)	*Phomopsis lithocarpus* (FS508)	Marine sediment	Indian Ocean	65
Tenellone J (78)	*Phomopsis lithocarpus* (FS508)	Deep Sea sediment	Indian Ocean	66
Tenellone L (79)	*Phomopsis lithocarpus* (FS508)	Deep Sea sediment	Indian Ocean	66
Pestalone (80)	*Pestalotia* sp. (CNL-365)	*Rosenvingea* sp. (brown alga, Scytosiphonaceae)	Bahamas Islands	67
	*Pestalotiopsis* sp. (ZJ-2009-7-6)	Soft coral	South China Sea, China	68
	*Pestalotiopsis* sp.	*Melia azedarach* (stem bark, Meliaceae)	Nanjing, Jiangsu, China	26
	*Pestalotiopsis neglecta* (F9D003)	Marine sediment	Shore of Gageo, Korea	69
Pestalone B (81)	*Pestalotiopsis neglecta* (F9D003)	Marine sediment	Shore of Gageo, Korea	69
Pestalone C (82)	*Pestalotiopsis neglecta* (F9D003)	Marine sediment	Shore of Gageo, Korea	69
Pestalone D (83)	*Pestalotiopsis neglecta* (F9D003)	Marine sediment	Shore of Gageo, Korea	69
Pestalone E (84)	*Pestalotiopsis neglecta* (F9D003)	Marine sediment	Shore of Gageo, Korea	69
Pestalone F (85)	*Pestalotiopsis neglecta* (F9D003)	Marine sediment	Shore of Gageo, Korea	69
Pestalone G (86)	*Pestalotiopsis neglecta* (F9D003)	Marine sediment	Shore of Gageo, Korea	69
Pestalone H (87)	*Pestalotiopsis neglecta* (F9D003)	Marine sediment	Shore of Gageo, Korea	69
FD549 (88)	*Talaromyces cellulolyticus* (BF-0307)	Soil sample	Meguro-ku, Tokyo, Japan	70
Penibenzone A (89)	*Penicillium purpurogenum* (IMM003)	*Edgeworthia chrysantha* (leaves, Thymelaeaceae)	Hangzhou Bay, Hangzhou, Zhejiang, China	71
Penibenzone B (90)	*Penicillium purpurogenum* (IMM003)	*Edgeworthia chrysantha* (leaves, Thymelaeaceae)	Hangzhou Bay, Hangzhou, Zhejiang, China	71
Arugosin H (91)	*Emericella nidulans* var. *acristata*	Marine green alga	Sardinia, Italy, Mediterranean Sea	72
	*Aspergillus nidulans* (FGSC A4)	—	Marburg, Germany	46
Arugosin I (92)	*Aspergillus nidulans* (FGSC A4)	—	Marburg, Germany	46
19-*O*-Methyl-22-methoxypre-shamixanthone (93)	*Mericella variecolor* (XSA-07-2)	*Cinachyrella* sp. (sponge, Tetillidae)	Yongxin Island, South China Sea	73
Pre-Shamixanthone (94)	*Mericella variecolor* (XSA-07-2)	*Cinachyrella* sp. (sponge, Tetillidae)	Yongxin Island, South China Sea	73
Chryxanthone A (95)	*Penicillium chrysogenum* (AD-1540)	*Grateloupia turuturu* (red alga, Halymeniaceae)	Qingdao, China	74
Chryxanthone B (96)	*Penicillium chrysogenum* (AD-1540)	*Grateloupia turuturu* (red alga, Halymeniaceae)	Qingdao, China	74
Pestalotinone B (97)	*Pestalotiopsis trachicarpicola* (SCJ551)	*Blechnum orientale* (stem, Blechnaceae)	Shatoujiao forestry center, Shenzhen, Guangdong, China	57
Pestalotinone C (98)	*Pestalotiopsis trachicarpicola* (SCJ551)	*Blechnum orientale* (stem, Blechnaceae)	Shatoujiao forestry center, Shenzhen, Guangdong, China	57
Pestalachloride B (99)	*Pestalotiopsis adusta* (L416)	Stem of an unidentified tree	Xinglong, Hainan, China	75
	*Pestalotiopsis* sp. (ZJ-2009-7-6)	Soft coral	South China Sea, China	68
	*Pestalotiopsis heterocornis*	*Phakellia fusca* (sponge, Bubaridae)	Xisha Islands, China	76
	*Pestalotiopsis neglecta* (F9D003)	Marine sediment	Shore of Gageo, Korea	69
Cephalanone D (100)	*Graphiopsis chlorocephala*	*Paeonia lactiflora* (leaves, Paeoniaceae)	Tohoku University, Japan	47
Cephalanone E (101)	*Graphiopsis chlorocephala*	*Paeonia lactiflora* (leaves, Paeoniaceae)	Tohoku University, Japan	47
Tenellone I (102)	*Diaporthe lithocarpus* (A740)	*Morinda officinalis* (twigs, Rubiaceae)	Gaoyao, Guangdong, China	77
Tenellone K (103)	*Phomopsis lithocarpus* (FS508)	Deep sea sediment	Indian Ocean	66
Tenellone M (104)	*Phomopsis lithocarpus* (FS508)	Deep sea sediment	Indian Ocean	66
Arugosin A (105)	*Aspergillus rugulosus* (I.M.I. 84338)	Wild	—	78
	*Emericella nidulans* var. *acristata*	Marine green alga	Sardinia, Italy, Mediterranean Sea	72
	*Aspergillus* nidulans (FGSC A4)	—	Marburg, Germany	46
Arugosin B (106)	*Aspergillus rugulosus* (I.M.I. 84338)	Wild	—	78
	*Emericella nidulans* var. *acristata*	Marine green alga	Sardinia, Italy, Mediterranean Sea	72
	*Aspergillus nidulans* (FGSC A4)	—	Marburg, Germany	46
Arugosin C (107)	*Aspergillus rugulosus* (A.R.M. 325)	Wild	—	79
Arugosin G (108)	*Emericella nidulans* var. *acristata*	Marine green alga	Sardinia, Italy, Mediterranean Sea	72
Balanol (109)	*Verticillium balanoides*	*Pinus palustris* needle litter (Pinaceae)	Near Hoffman, North Carolina, USA	80
Cytosporin A (110)	*Cytospora rhizophorae* (A761)	*Morinda officinalis* (twigs, Rubiaceae)	Gaoyao, Guangdong, China	81
Cytosporin B (111)	*Cytospora rhizophorae* (A761)	*Morinda officinalis* (twigs, Rubiaceae)	Gaoyao, Guangdong, China	81
Cytosporin C (112)	*Cytospora rhizophorae* (A761)	*Morinda officinalis* (twigs, Rubiaceae)	Gaoyao, Guangdong, China	81
Cytosporin D (113)	*Cytospora rhizophorae* (A761)	*Morinda officinalis* (twigs, Rubiaceae)	Gaoyao, Guangdong, China	81
Cytorhizin A (114)	*Cytospora rhizophorae* (A761)	*Morinda officinalis* (twigs, Rubiaceae)	Gaoyao, Guangdong, China	82
Cytorhizin B (115)	*Cytospora rhizophorae* (A761)	*Morinda officinalis* (twigs, Rubiaceae)	Gaoyao, Guangdong, China	82
Cytorhizin C (116)	*Cytospora rhizophorae* (A761)	*Morinda officinalis* (twigs, Rubiaceae)	Gaoyao, Guangdong, China	82
Cytorhizin D (117)	*Cytospora rhizophorae* (A761)	*Morinda officinalis* (twigs, Rubiaceae)	Gaoyao, Guangdong, China	82
Cytorhizophin A (118)	*Cytospora rhizophorae* (A761)	*Morinda officinalis* (twigs, Rubiaceae)	Gaoyao, Guangdong, China	54
Cytorhizophin B (119)	*Cytospora rhizophorae* (A761)	*Morinda officinalis* (twigs, Rubiaceae)	Gaoyao, Guangdong, China	54
Cytorhizophin J (120)	*Cytospora heveae* (NSHSJ-2)	*Sonneratia caseolaris* (stem, Lythraceae)	Nansha Mangrove National Nature Reserve in Guangdong, China	83
Delicoferone A (121)	*Delitschia confertaspora* (ATCC 74209)	*Procavia capensis* (Dung of a rock hyrax, Procaviidae)	Dassie, Namibia	84
Delicoferone B (122)	*Delitschia confertaspora* (ATCC 74209)	*Procavia capensis* (Dung of a rock hyrax, Procaviidae)	Dassie, Namibia	84
Acremonidin A (123)	*Acremonium* sp. (LL-Cyan 416)	—	—	45
	*Hypocreales* (MSX 17022)	Leaf litter from a beech tree community	Hillsborough, NC, USA	19
Acremonidin B (124)	*Acremonium* sp. (LL-Cyan 416)	—	—	45
Acremonidin C (125)	*Acremonium* sp. (LL-Cyan 416)	—	—	45
	*Hypocreales* (MSX 17022)	Leaf litter from a beech tree community	Hillsborough, NC, USA	19
Acremonidin D (126)	*Acremonium* sp. (LL-Cyan 416)	—	—	45
Guignasulfide (127)	*Guignardia* sp. (IFB-E028)	*Hopea hainanensis* (leaves, Dipterocarpaceae)	Hainan Island, China	36
	*Aspergillus fumigatus*	*Solanum insanum* (fruit, Solanaceae)	Central Province of Sri Lanka	37
Microsphaerin A (128)	*Microsphaeropsis* sp. (F2076 and F2078)	Lake sediment	Singapore	85
Microsphaerin D (129)	*Microsphaeropsis* sp. (F2076 and F2078)	Lake sediment	Singapore	85
Phomalevone B (130)	*Phoma* sp. (MYC-1734 = NRRL 39060)	Montane dry forest (Ohi'a)	Koloko Hue Street, Kailua-Kona, Hawaii Co., HI	86
Orbiocrellone A (131)	*Orbiocrella petchii* (BCC 51377)	A scale-insect (Hemiptera) underside of a leaf (Poaceae)	Chae Son National Park, Lampang, Thailand	53
Orbiocrellone B (132)	*Orbiocrella petchii* (BCC 51377)	A scale-insect (Hemiptera) underside of a leaf (Poaceae)	Chae Son National Park, Lampang, Thailand	53
Orbiocrellone C (133)	*Orbiocrella petchii* (BCC 51377)	A scale-insect (Hemiptera) underside of a leaf (Poaceae)	Chae Son National Park, Lampang, Thailand	53
Orbiocrellone D (134)	*Orbiocrella petchii* (BCC 51377)	A scale-insect (Hemiptera) underside of a leaf (Poaceae)	Chae Son National Park, Lampang, Thailand	53
Orbiocrellone E (135)	*Orbiocrella petchii* (BCC 51377)	A scale-insect (Hemiptera) underside of a leaf (Poaceae)	Chae Son National Park, Lampang, Thailand	53
Digriseophene A (136)	*Penicillium* sp. (ct-28)	*Corydlis tomentella* (leaves, Papaveraceae)	Jinfo Mountain, Chongqing, China	60
Dipleosporone A (137)	*Pleosporales* sp. (YY-4)	*Uncaria rhynchophylla* (plant, Rubiaceae)	Jian, Jiangxi, China	63
Dipleosporone B (138)	*Pleosporales* sp. (YY-4)	*Uncaria rhynchophylla* (plant, Rubiaceae)	Jian, Jiangxi, China	63
Dipleosporone C (139)	*Pleosporales* sp. (YY-4)	*Uncaria rhynchophylla* (plant, Rubiaceae)	Jian, Jiangxi, China	63
Acredinone A (140)	*Acremonium* sp. (F9A015)	*Suberites japonicus* (sponge, Suberitidae)	Ga-geo Island near the southwest sea of Korea	51
Acredinone B (141)	*Acremonium* sp. (F9A015)	*Suberites japonicus* (sponge, Suberitidae)	Ga-geo Island near the southwest sea of Korea	51
Acredinone C (142)	*Acremonium* sp. (F9A015)	*Suberites japonicus* (sponge, Suberitidae)	Ga-geo Island near the southwest sea of Korea	87
Celludinone B (143)	*Talaromyces cellulolyticus* (BF-0307)	Soil sample	Meguro-ku, Tokyo, Japan	70
Ent-secalonic acid I (144)	*Orbiocrella petchii* (BCC 51377)	A scale-insect (Hemiptera) underside of a leaf (Poaceae)	Chae Son National Park, Lampang, Thailand	53
Griseophenexanthone A (145)	*Penicillium* sp. (ct-28)	*Corydlis tomentella* (leaves, Papaveraceae)	Jinfo Mountain, Chongqing, China	60
Asperphenin A (146)	*Aspergillus* sp. (F452)	Submerged decaying wood	Shore of Jeju Island, Korea	88
Asperphenin B (147)	*Aspergillus* sp. (F452)	Submerged decaying wood	Shore of Jeju Island, Korea	88

a *, ** Same nomenclature but different structures.

## 2 Research methodology

Reviewing of literature was carried out through online search on ScienceDirect, Wiley Online Library, SCOPUS, Google Scholar, PubMed, Taylor & Francis, Springer, Bentham, Thieme, and JACS. The data was retrieved using “Benzophenones + Fungi”, OR “Benzophenones + Biological activity” OR “Benzophenones + Biosynthesis” as keywords. All studies that reported the isolation, structural characterization, biosynthesis, and bioactivities of fungal BPs, as well as reviews and book chapters were included. The peer-reviewed journals' English language published papers from 1963 to 2023 were included. Included studies were assessed through reading their titles, abstracts, and full texts. The no full access (*e.g.*, conference proceedings), irrelevant, and non-reviewed journals published work were excluded. For the non-English paper, the information was extracted from the English abstracts. The reported works on BPs from other sources were not included. In the current review, a total of 110 references were discussed.

## 3 Biological activities of benzophenones

Various benzophenones derivatives have been isolated from fungi obtained from different extracts using diverse chromatographic techniques and elucidated by different spectral analyses as well as Xray, CD, ECD, and chemical methods. These metabolites have been assessed for different bioactivities that have been summarized here.

### 3.1. Plant growth inhibitory and anti-nematode activities

**Fig. 2:**
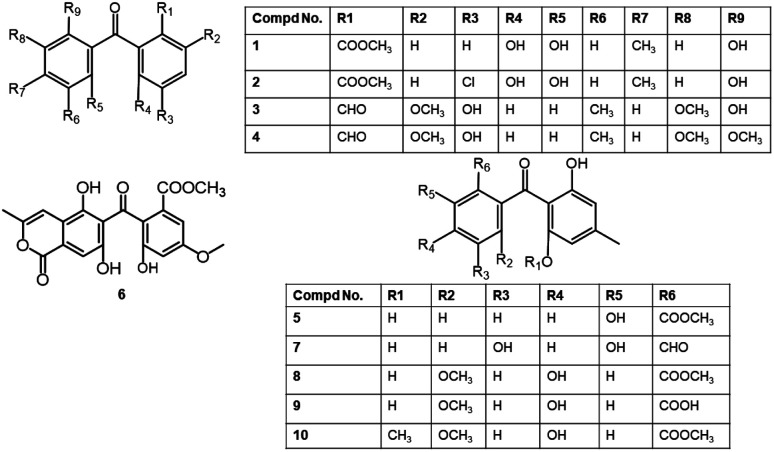
Structures of benzophenones 1–10.

Hashimoto *et al.* purified and characterized compounds 3 and 4 from the EtOAc extract of *Daldinia concentrica* using NMR, Xray, and chemical degradation (Fig. 2). These metabolites at 5 ppm completely prohibited rice root germination in husk.^23^ Also, 8 exhibited moderate (LD_90_ 50 ppm) anti-nematode potential *versus Caenorhabditis elegans* and inhibited germination of cress seeds at 100 ppm.^27^

### 3.2. Antimicrobial, anti-mycobacterial, and antialgal activities

The microbe's resistance to the available antibiotics becomes the main health concern. Therefore, there is a pressing requirement for finding out new types of antimicrobials with unfamiliar mechanisms to overcome multidrug-resistant microbe infections.^89^

**Fig. 3:**
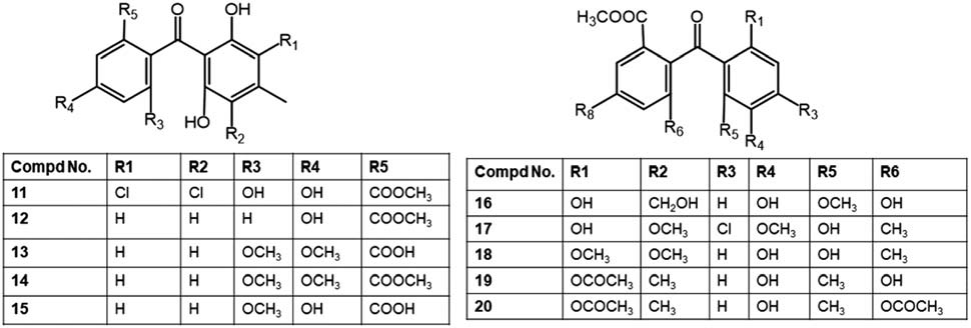
Structures of benzophenones 11–20.

Sulochrin (8) and demethylsulochrin (9) were separated from the leaf litters-derived *Aspergillus* species EtOAc extract by SiO_2_ CC. Compound 97 had no antimicrobial capacity *versus E. coli* or phyto-pathogens: *Rhizoctonia solani* and *Gaeumannomyces graminis* var *tritici* (Conc. < 200 ppm).^27^ Two new compounds: penibenzophenones A and B (17 and 18), along with 8 were isolated from the EtOAc extract of *Bruguiera sexangula* var. *rhynchopetala*-harbouring *Penicillium citrinum* (HL-5126) fermentation broth. Their structures were elucidated by extensive NMR, MS, and X-ray analyses (Fig. 3). Compound 17 is an example of chlorinated benzophenones. Among these metabolites, 17 revealed weak antibacterial effectiveness *versus S. aureus* (MIC 20 μg mL^−1^).^31^

**Fig. 4:**
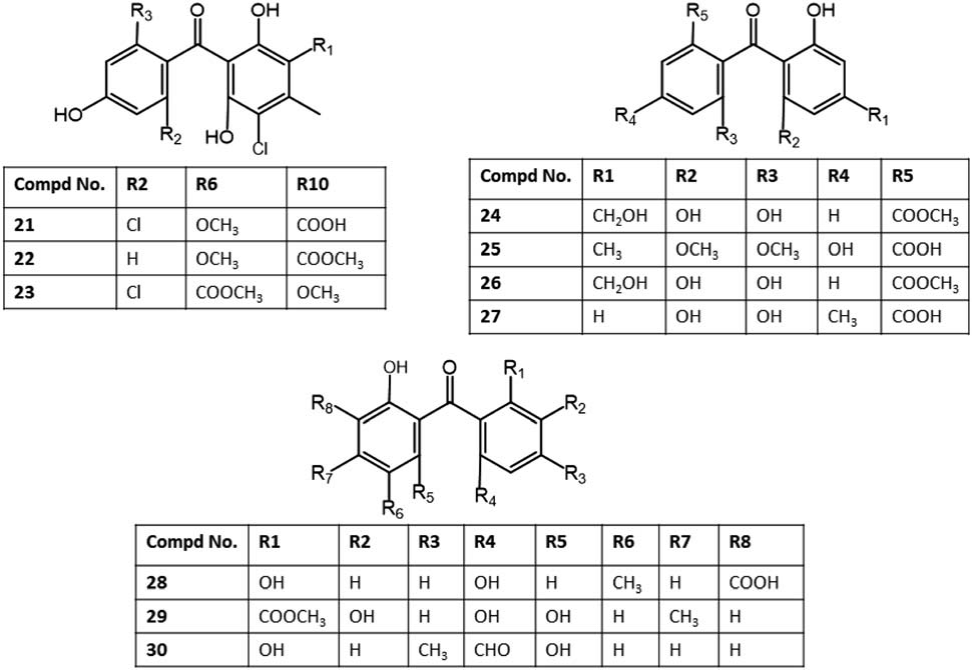
Structures of benzophenones 21–30.

Additionally, the new benzophenone derivatives: penibenzophenones C (19) and D (20), together with 8 and 16 were separated by SiO_2_/Sephadex LH-20/HPLC from the EtOAc extract of *Penicillium* sp. isolated *Acanthus ilicifolius* collected from the South China Sea and elucidated by NMR and MS analyses. Compounds 19 and 20 demonstrated antibacterial efficacy *versus* MRSA (MICs 3.12 and 6.25 μg mL^−1^, respectively), compared to ciprofloxacin (MIC 1.56 μg mL^−1^), whilst 8 and 16 had weak activity in the microplate assay method (Table 2).^32^ Compounds 22 and 23 isolated from *Aspergillus flavipes* DL11 were assessed for antibacterial potential against *S. aureus* (ATCC-43300, ATCC-29213, ATCC-33591, and ATCC-25923), *E. faecalis* ATCC-51299, *E. faecalis* ATCC-35667, and *V. parahaemolyticus* ATCC-17802 in the broth microdilution (Fig. 4). Interestingly, 22 revealed powerful inhibitory potential *versus* all *S. aureus* strains (MICs 1.56 to 12.5 μg mL^−1^) and moderate potential *versus E. faecalis* ATCC-51299 and ATCC-35667 (MICs 50 and 100 μg mL^−1^, respectively). On the other hand, 23 had a potent antibacterial capacity *versus* all tested strains (MICs 1.56 to 12.5 μg mL^−1^) except *V. parahaemolyticus* ATCC-17802, compared to vancomycin HCl and ampicillin sodium.^39^

**Table 2:** Antibacterial activity of the reported fungal benzophenones^a^

Compd no	Assay/bacterial strain	Biological results		Ref.
		Compound	Positive control	
10	Agar dilution/*H. pylori*	10.0 μg mL^−1^*	Ampicillin 2.0 μg mL^−1^*	35
		28.9 μM*	Ampicillin 5.4 μM*	36
19	Microplate/MRSA	3.12 μg mL^−1^*	Ciprofloxacin 1.56 μg mL^−1^*	32
	Microplate/*S. aureus*	6.25 μg mL^−1^*	Ciprofloxacin 0.39 μg mL^−1^*	32
20	Microplate/MRSA	6.25 μg mL^−1^*	Ciprofloxacin 1.56 μg mL^−1^*	32
	Microplate/*S. aureus*	12.5 μg mL^−1^*	Ciprofloxacin 0.39 μg mL^−1^*	32
22	Broth microdilution/*S. aureus* (ATCC43300)	12.5 μg mL^−1^*	Vancomycin HCl 1.56 μg mL^−1^*	39
			Ampicillin sodium 25.0 μg mL^−1^*	
	Broth microdilution/*S. aureus* (ATCC29213)	3.13 μg mL^−1^*	Vancomycin HCl 0.78 μg mL^−1^*	39
			Ampicillin sodium 6.25 μg mL^−1^*	
	Broth microdilution/S. aureus (ATCC33591)	1.56 μg mL^−1^*	Vancomycin HCl 1.56 μg mL^−1^*	39
			Ampicillin sodium 25.0 μg mL^−1^*	
	Broth microdilution/*S. aureus* (ATCC25923)	1.56 μg mL^−1^*	Vancomycin HCl 3.13 μg mL^−1^*	39
			Ampicillin sodium 0.78 μg mL^−1^*	
	Broth microdilution/*E. faecalis* (ATCC51299)	50.0 μg mL^−1^*	Vancomycin HCl 25.0 μg mL^−1^*	39
			Ampicillin sodium 25.0 μg mL^−1^*	
23	Broth microdilution/*S. aureus* (ATCC43300)	6.25 μg mL^−1^*	Vancomycin HCl 1.56 μg mL^−1^*	39
			Ampicillin sodium 25.0 μg mL^−1^*	
	Broth microdilution/*S. aureus* (ATCC29213)	3.13 μg mL^−1^*	Vancomycin HCl 0.78 μg mL^−1^*	39
			Ampicillin sodium 6.25 μg mL^−1^*	
	Broth microdilution/*S. aureus* (ATCC33591)	1.56 μg mL^−1^*	Vancomycin HCl 1.56 μg mL^−1^*	39
			Ampicillin sodium 25.0 μg mL^−1^*	
	Broth microdilution/*S. aureus* (ATCC25923)	1.56 μg mL^−1^*	Vancomycin HCl 3.13 μg mL^−1^*	39
			Ampicillin sodium 0.78 μg mL^−1^*	
	Broth microdilution/*E. faecalis* (ATCC51299)	12.5 μg mL^−1^*	Vancomycin HCl 25.0 μg mL^−1^*	39
			Ampicillin sodium 25.0 μg mL^−1^*	
	Broth microdilution/*E. faecalis* (ATCC35667)	12.5 μg mL^−1^*	Vancomycin HCl 3.13 μg mL^−1^*	39
			Ampicillin sodium 6.25 μg mL^−1^*	
25	Agar dilution/*H. pylori*	25.0 μg mL^−1^*	Ampicillin 2.0 μg mL^−1^*	35
		60.2 μM*	Ampicillin 5.4 μM*	36
28	Disk diffusion/*A. hydrophilia*	8.0 μg mL^−1^*	Chloromycetin 4 μg mL^−1^*	43
37	Microplate/*E. coli*	4.0 μg mL^−1^*	Chloramphenicol 1.0 μg mL^−1^*	48
	Microplate/*P. aeruginosa*	4.0 μg mL^−1^*	Chloramphenicol 4.0 μg mL^−1^*	48
	Microplate/*S. aureus*	8.0 μg mL^−1^*	Chloramphenicol 2.0 μg mL^−1^*	48
	Microplate/*Vibrio alginolyticus*	4.0 μg mL^−1^*	Chloramphenicol 0.5 μg mL^−1^*	48
	Microplate/*V. harveyi*	8.0 μg mL^−1^*	Chloramphenicol 2.0 μg mL^−1^*	48
	Microplate/*V. parahaemolyticus*	4.0 μg mL^−1^*	Chloramphenicol 2.0 μg mL^−1^*	48
68	Alamar Blue/*S. aureus*	10.0 μg mL^−1^**	Kanamycin 1.25 μg mL^−1^**	57
	Alamar Blue/MRSA	10.0 μg mL^−1^**	Vancomycin 0.625 μg mL^−1^**	57
	Alamar Blue/VSE	10.0 μg mL^−1^**	Vancomycin 1.25 μg mL^−1^**	57
	Alamar Blue/VRE	>10.0 μg mL^−1^**	Vancomycin >40.0 μg mL^−1^**	57
80	Serial dilution/MRSA (31956)	12.5 μM*	Rifampin 0.03 μM*	68
	Serial dilution/MRSA (30740)	6.25 μM*	Rifampin 0.0037 μM*	68
	Serial dilution/MRSA (31709)	12.5 μM*	Rifampin 0.0074 μM*	68
	Serial dilution/MRSA (31007)	12.5 μM*	Rifampin 0.0009 μM*	68
	Serial dilution/MRSA (31692)	12.5 μM*	Rifampin 0.0037 μM*	68
	Serial dilution/*B. megaterium*	0.078 μM*	Ciprofloxacin 0.312 μM*	68
	Serial dilution/*M. lysodeikticus*	6.25 μM*	Ciprofloxacin 3.125 μM*	68
	Broth microdilution/*E. coli*	3.2 μg mL^−1^**	Streptomycin 0.7 μg mL^−1^**	26
	Broth microdilution/*P. aeruginosa*	6.5 μg mL^−1^**	Streptomycin 1.0 μg mL^−1^**	26
	Broth microdilution/*S. aureus*	5.0 μg mL^−1^**	Penicillin 1.2 μg mL^−1^**	26
	Broth microdilution/*C. glabrata*	2.6 μg mL^−1^**	Amphotericin B 0.2 μg mL^−1^**	26
	Alamar Blue/*S. aureus*	5.0 μg mL^−1^**	Kanamycin 1.25 μg mL^−1^**	57
	Alamar Blue/MRSA	5.0 μg mL^−1^**	Vancomycin 0.625 μg mL^−1^**	57
	Alamar Blue/VSE	2.5 μg mL^−1^**	Vancomycin 1.25 μg mL^−1^**	57
	Alamar Blue/VRE	>10.0 μg mL^−1^**	Vancomycin >40.0 μg mL^−1^**	57
84	Alamar Blue/*S. aureus*	5.0 μg mL^−1^**	Kanamycin 1.25 μg mL^−1^**	57
	Alamar Blue/MRSA	10.0 μg mL^−1^**	Vancomycin 0.625 μg mL^−1^**	57
	Alamar Blue/VSE	5.0 μg mL^−1^**	Vancomycin 1.25 μg mL^−1^**	57
	Alamar Blue/VRE	>10.0 μg mL^−1^**	Vancomycin >40.0 μg mL^−1^**	57
85	Alamar Blue/*S. aureus*	10.0 μg mL^−1^**	Kanamycin 1.25 μg mL^−1^**	57
	Alamar Blue/MRSA	10.0 μg mL^−1^**	Vancomycin 0.625 μg mL^−1^**	57
	Alamar Blue/VSE	10.0 μg mL^−1^**	Vancomycin 1.25 μg mL^−1^**	57
	Alamar Blue/VRE	>10.0 μg mL^−1^**	Vancomycin >40.0 μg mL^−1^**	57
99	Micro broth dilution/*B. subtilis*	3.0 μg mL^−1^*	Ciprofloxacin 0.25 μg mL^−1^*	76
	Micro broth dilution/*S. aureus*	3.0 μg mL^−1^*	Ciprofloxacin 0.13 μg mL^−1^*	76
	Alamar Blue/*S. aureus*	2.5 μg mL^−1^**	Kanamycin 1.25 μg mL^−1^**	57
	Alamar Blue/MRSA	1.25 μg mL^−1^**	Vancomycin 0.625 μg mL^−1^**	57
	Alamar Blue/VSE	5.0 μg mL^−1^**	Vancomycin 1.25 μg mL^−1^**	57
	Alamar Blue/VRE	10.0 μg mL^−1^**	Vancomycin >40.0 μg mL^−1^**	57
127	Agar dilution/*H. pylori*	42.9 μM*	Ampicillin 5.4 μM*	36
129	Agar dilution/*S. aureus*	1.3 μM***	—	85
	Agar dilution/MRSA	1.0 μM***	—	85
	Agar dilution/*E. faecalis*	1.3 μM***	—	85
	Agar dilution/*S. pneumoniae*	3.6 μM***	—	85
	Agar dilution/*B. subtilis*	3.0 μM***	—	85
	Agar dilution/*M. catarrhalis*	1.3 μM***	—	85

a *MIC; ** MIC_50_; ***IC_90_; ****IC_50_; VRE: Vancomycin-resistance *E. faecium*; VSE: Vancomycin-sensitive *E. faecium*.

Ma *et al.* reported the separation of rhizoctonic acid (25), a new benzophenone derivative and the formerly reported analogue 10 from the culture of *Rhizoctonia* sp. Cy064 associated with *Cynodon dactylon* leaf that were elucidated using various spectral analyses. These metabolites were *in vitro* assessed of their antibacterial potential *versus Helicobacter pylori*, including 5 clinically isolated and one reference ATCC 43504 strains in the agar dilution method. These compounds showed antibacterial influence *versus* all tested strains (MICs 25.0 to 10.0 μg mL^−1^) compared to ampicillin (MIC 2.0 μg mL^−1^).^35^

Investigation of *Penicillium* sp. MA-37 harboring *ruguiera gymnorrhiza* led to separation of a new benzophenone; iso-monodictyphenone (28), in addition to 27 from the EtOAc extract using SiO_2_/Sephadex LH-20/PR-18 CC and preparative TLC. Compound 28 differed from 27 mainly in the positions of ring A substituents. Compound 28 demonstrated antibacterial efficacy *versus Aeromonas hydrophilia* (MIC 8 μg mL^−1^) in comparison to chloromycetin (MIC 4 μg mL^−1^).^43^

**Fig. 5:**
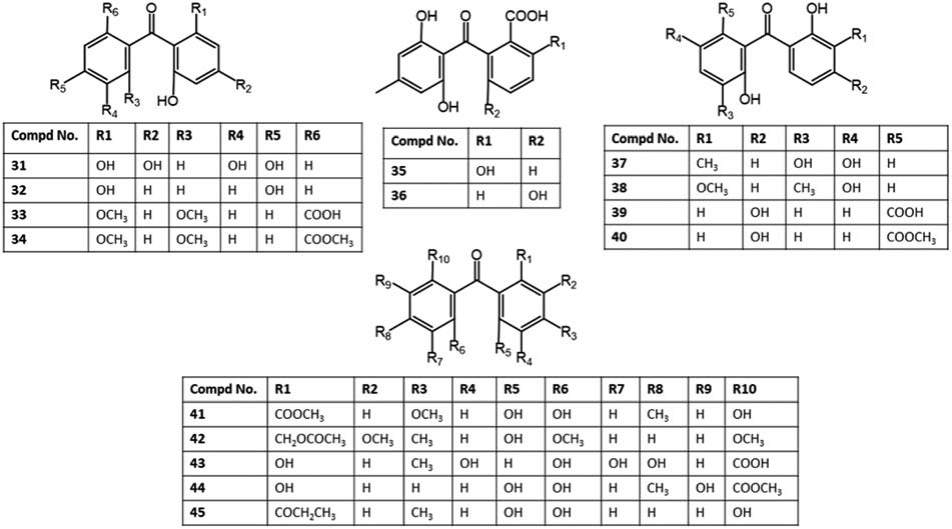
Structures of benzophenones 31–45.

Two new benzophenone derivatives; 37 and 38 were isolated from the EtOAc extract of *Laurencia okamurai-associated Talaromyces islandicus* EN-501 by SiO_2_/Sephadex LH-20 CC and HPLC and assigned by NMR and X-ray analyses (Fig. 5).

Compound 37 revealed potent effectiveness *versus* human pathogens; *E. coli*, *P. aeruginosa*, and *S. aureus* and aquatic bacteria; *Vibrio alginolyticus*, *V. harveyi*, and *V. parahaemolyticus* (MICs ranged from 4.0 to 8.0 μg mL^−1^) in the microplate assay, however, 38 had weak potential *versus* the tested strain (MIC > 64 μg mL^−1^) in comparison to chloramphenicol (MICs ranged from 0.5 to 4 μg mL^−1^), indicating that the C-3 methoxylation weakened the activity (37*vs.*38).^48^*Diaporthe* sp. SYSU.HQ3 yielded tenellone C (71) that possessed inhibitory potential *versus* MptpB (*Mycobacterium tuberculosis* protein tyrosine phosphatase B) (IC_50_ 5.2 μM).^90^

Pestalone (80), a new antibiotic derivative was biosynthesized by the brown alga *Rosenvingea* sp. associated *Pestalotia* sp. in a mixed fermentation with an antibiotic-resistant unidentified marine bacterium CNL-365. Besides, this compound was not produced by the individual strains, suggesting its fungal production is boosted by bacterial competition. It was isolated by RP-18/Sephadex LH-20/SiO_2_ CC and assigned using spectral, chemical, and Xray analyses. It featured a di-chlorinated benzene moiety. Interestingly, 80 possessed potent antibacterial potential *versus* vancomycin-resistant *Enterococcus faecium* (VREF) and MRSA (methicillin-resistant *S. aureus*) (MICs 78 and 37 ng mL^−1^, respectively) that should be further assessed in more advanced models of infectious disease.^67^ Furthermore, chromatographic separation of *Pestalotiopsis* sp. ZJ.2009.7.6`s EtOAc extract using SiO_2_ and Sephadex LH-20 CC yielded 80 and 99 that were established by NMR tools. Compound 80 exhibited selective and moderate capacities *versus* various MRSA-31007, 30740, 31709, 31692, 31956) (MICs 6.25–12.5 μM) compared to rifampin (0.0009–0.03 μM), however, its structure-related analogue 99 had weak efficacy *versus S. aureus* in a serial dilution technique using 96-well microtiter plates. On the other side, only 80 possessed selective potential *versus Micrococcus lysodeikticus* and *B. megaterium* (MICs 6.25 and 0.078 μM, respectively), comparing to ciprofloxacin (MICs 3.125 and 0.312 μM, respectively), indicating that the methoxy or aldehyde group influenced the activity.^68^ In another study by Li *et al.*, 99 reported from *Pestalotiopsis adusta* was found to have significant effectiveness *versus* plant pathogens; *Verticillium aibo-atrum, Fusarium culmorum*, and *Gibberella zeae* (MICs 7.9, 4.7, and 1.1 μM, respectively).^75^ Besides, it displayed antibacterial efficacy *versus S. aureus* and *B. subtilis* (MICs 3.0 μg mL^−1^) relative to ciprofloxacin (MICs 0.13 and 0.25 μg mL^−1^, respectively).^76^ In 2017, Song *et al.* purified 7 as a new benzophenone, alongside with 80 from solid cultures EtOAc extract of *Pestalotiopsis* sp. inhabited *Melia azedarach* utilizing SiO_2_/Sephadex LH-20 CC and preparative RP-HPLC. They were investigated for antimicrobial capacity *versus B. subtilis* ATCC6633, *S. aureus* ATCC25923, *E. coli* ATCC25922, *P. aeruginosa* ATCC9027, *C. glabrata* ATCC90030 in the broth microdilution method. Compound 80 also demonstrated remarkable activity *versus C. glabrata* (MIC_50_ 2.6 μg mL^−1^).^26^ In 2022, Jiang *et al.* separated new pestalone-related benzophenones; 51, 97, and 98, along with 68, 80, 84, 85, and 99 from *Pestalotiopsis trachicarpicola* SCJ551 culture EtOAc extract using SiO_2_/RP-18/Sephadex LH-20 CC and HPLC that were established by spectroscopic analyses.^57^ Compounds 51 and 97–99 had activity *versus S. aureus* ATCC-6548, MRSA, *Enterococcusfaecium*, and vancomycin-resistance *E. faecium* (MICs 1.25–10.0 μg mL^−1^). It was revealed that the C-14 aldehyde reduction into oxymethyl increased the activity. Also, the chlorination slightly increased the antibacterial potential (85*vs.*80 and 84*vs.*68).^57^ The new metabolites: acremonidins A–E (29 and 122–125) purified from the MeOH extract of *Acremonium* sp. LL-Cyan 416 by RP-18 CC and HPLC possessed moderate antibiotic activity *versus* MRS and VRE (vancomycin-resistant *Enterococci*) (MICs ranging from 8.0 to 64.0 μg mL^−1^) in the broth dilution method, whereas 122 was the most active (MICs 8.0–32.0 μg mL^−1^). The C-6 acetyl group was important for retaining potency (122*vs.*123).^45^

**Scheme 1:**
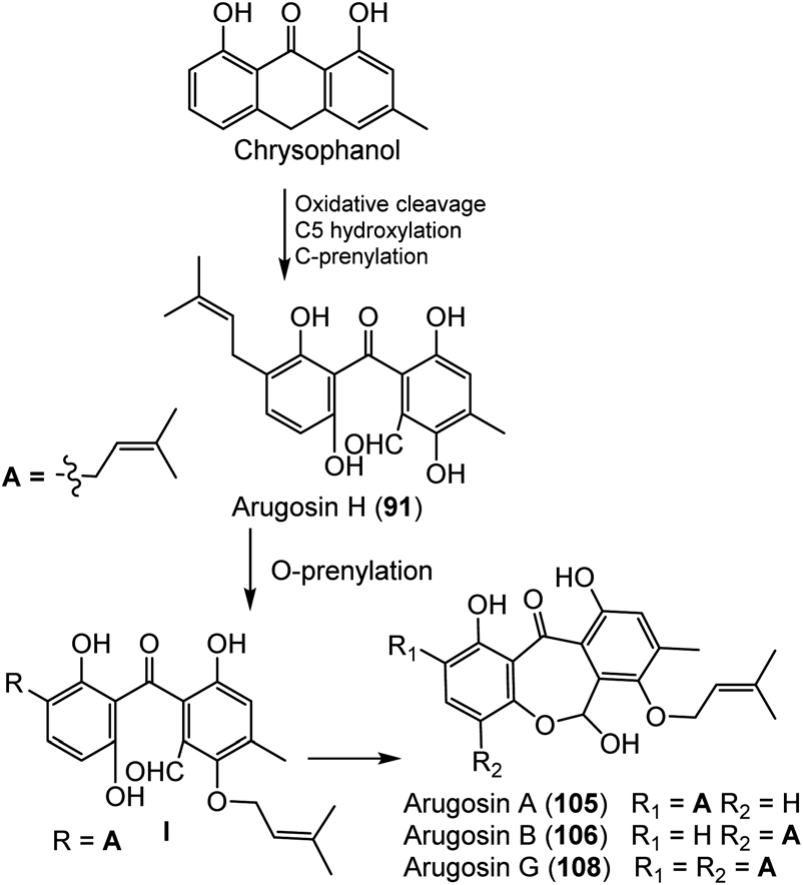
Biosynthetic pathway of compounds 91, 105, 106, and 108 from chrysophanol.^72^

*Emericella nidulans* var. *acristata* obtained from a Mediterranean green alga yielded 91, 105, 106, and 108 that were purified from the culture EtOAc extract using SiO_2_/Sephadex LH-20 CC/HPLC. These metabolites were assessed for antifungal, antibacterial, and antialgal potential (Conc. 50 μg per disk) in the agar diffusion method. Compound 91 exhibited antifungal and antialgal potential *versus Mycotypha microspora* and *Chlorella fusca*, respectively (IZD 3.0 and 2.0 mm, respectively), whereas 105 and 106 (as a mixture) displayed antibacterial efficacy *versus Bacillus megaterium* (4.0 mm).^72^ Arugosin H (91) was proposed to be originated from an anthrone; chrysophanol that undergoes oxidative cleavage to give an aldehyde group, with subsequent hydroxylation and C-prenylation (Scheme 1). Further, the aldehyde group is converted to a hemiacetal function to produce the other tricyclic and prenylated metabolites 105, 106, and 108.^72^

From *Cytospora rhizophorae* A761 associated with *Morinda officinalis*, cytosporins A–D (110–113), novel benzophenone derivatives were separated. Compounds 110–113 are hemiterpenoid-benzophenone conjugated hetero-dimers, having an unrivalled eight/seven-membered ring system. Their structures were characterized based on spectroscopic, ECD, and Xray analyses. Their configuration was assigned as 2′*R* for 110 and 111, 7*R*/2′*R* for 112, and 7*S*/2′*R* for 113. These metabolites had no significant antibacterial potential *versus E. coli* and *S. aureus* even at Conc. 250 μg mL^−1^.^81^ From the same fungus, Liu *et al.* also reported the separation of cytorhizins A–D (114–117), novel polyketide heterodimers by SiO_2_/RP-18/Sephadex LH-20 CC and RP-HPLC. These compounds have uncommon 6/6/5/6/7 or 6/6/5/6/8 pentacyclic ring skeleton forming a fascinating cage-like skeleton, involving a highly substituted benzophenone scaffold and a poly-oxygenated isopentyl moieties that were assigned by spectroscopic and Xray analyses. These compounds possessed no notable effectiveness *versus S. aureus* CMCC-26003 and *E. coli* ATCC-8739 even at Conc. 100 μM.^82^

Further, Liu *et al.* identified a novel pair of enantiomeric hemiterpene-benzophenones; (+)/(−)-cytorhizophin A (118), as well as cytorhizophin B (119) that featured an unprecedented 6/7/6/7 tetracyclic fused ring system, in addition to related precursor 45 from *C. rhizophorae* A76.^54^ Their structures were assigned by spectroscopic, X-ray, and ECD. They had no antibacterial potential against *E. coli* and *S. aureus*.^54^

**Scheme 2:**
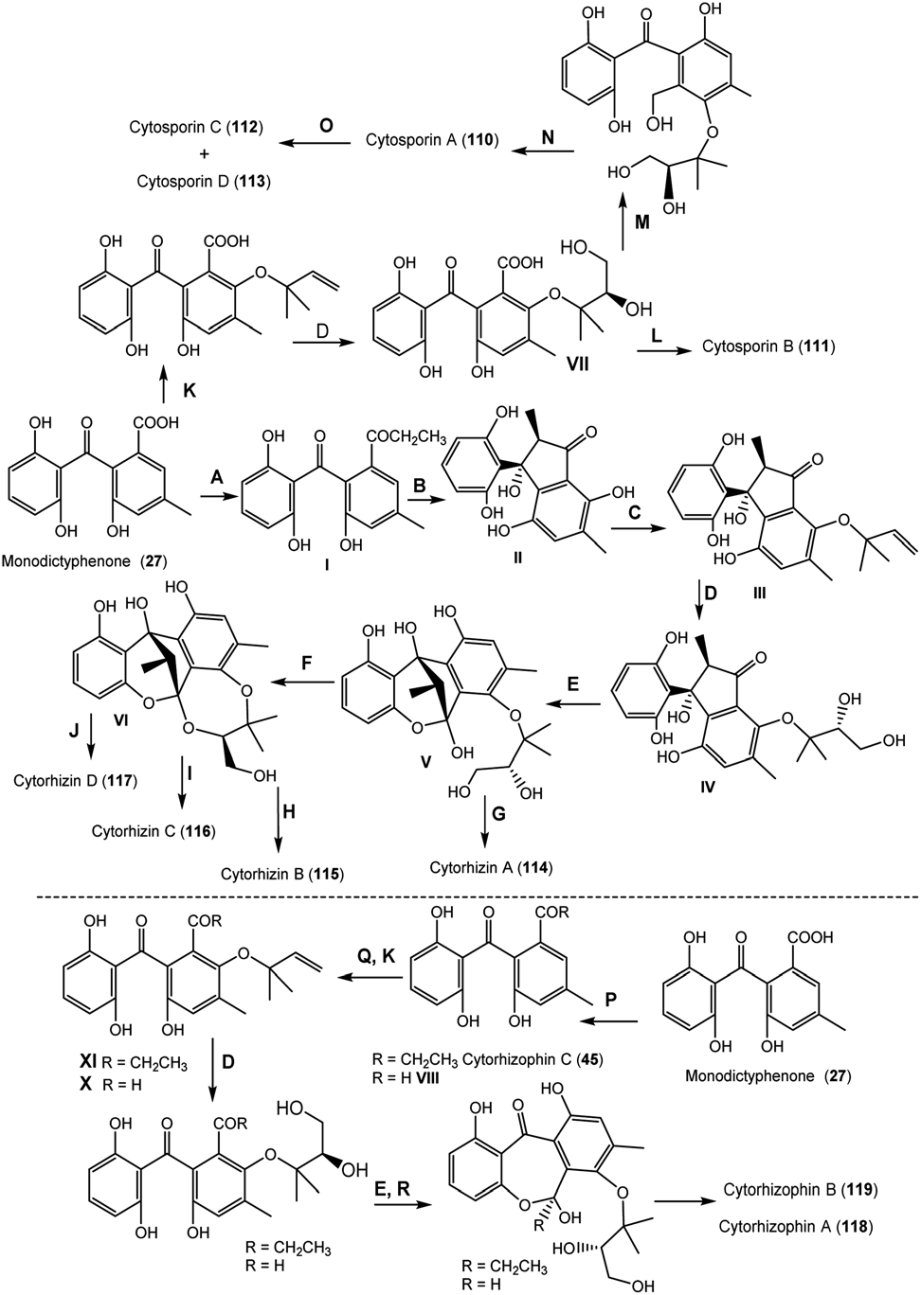
Biosynthetic pathway of 45 and 110–118 from monodictyphenone (27).^54,81,82^ A: Functionality transformation; B: aldol condensation; C: reverse prenylation; D: dihydroxylation; E: hemi-ketalization; F: C-1 OH ketalization; G: C-2 OH ketalization; H: chlorination; I: methylation; J: esterification; K: prenylation; L: intramolecular lactonization; M: carboxylic acid reduction; N: etherification; O: carbonyl reduction; P: reduction or functional group transformation; Q: oxidation; R: hemi-acetalization.

It was proposed that monodictyphenone (27) affords cytorhizophin C (45) and VIII. Further, the selective oxidation and prenylation generate hybrid intermediates IX and X, which then undergo cyclization with subsequent dihydroxylation/spontaneous ketalization to give 118 and 119 (Scheme 2). Also, 114–117 originate from 27 through series of reactions to install the propionyl moiety^54^ (Scheme 2). Then, aldol condensation and a sequence of reverse prenylation, dihydroxylation, and hemi-ketalization accomplish the cage-like benzophenol core, giving a precursor (V). Further, the regioselective ketalization in C-1 or C-2 free OH group results in 114 and VI, respectively. On the other side, 115–117 are produced from III through methylation, esterification, and chlorination, respectively.^82^ In the same manner, 110–113 are generated from precursor VII, which is formed from 27 by including hemiterpene nucleus through stereoselective dihydroxylation and chemo-selective prenylation. Its intramolecular lactonization results in 111, whereas 110 is produced from VII by carboxylic acid reduction and etherification. Further, 112 and 113 are generated from 110 by carbonyl reduction.^81^

Guignasulfide (127), a first S-having benzophenone dimer and the formerly reported 10 and 25 were separated from the culture of *Hopea hainanensis* leaves-accompanied *Guignardia* sp. utilizing SiO_2_/RP-18/Sephadex LH-20 CC. Compounds 10, 25, and 127 revealed moderate growth inhibition on *Helicobacter pylori* (MICs 28.9, 60.2, and 42.9 μM, respectively), compared to ampicillin (MIC 5.4 μM).^36^

Bioassay-directed separation using MRSA whole cell assay resulted in separation of two novel benzophenone dimers, microsphaerins A and D (128 and 129) from the soil-derived *Microsphaeropsis* sp. by HPLC. Their structures were elucidated using spectral and X-ray analyses. In the MRSA whole cell assay, 128 and 129 had antibacterial potential (IC_90_ 3 and 1 μM, respectively), therefore, 129 was further assessed *versus* Gram positive (*S. aureus* ATCC25923, MRSA ATCC33591, *E. faecalis* ATCC51299, *S. pneumoniae* ATCC 49619, and *B. subtilis* ATCC6633) and Gram negative (*E. coli* ATCC25922, *K pneumoniae* ATCC10031, *M. catarrhalis* ATCC49143, *H. influenzae* ATCC49247, and *P. aurogenosa* ATCC27853). Compound 129 was found to have notable effectiveness various Gram positive strains (IC_90_ ranged from 1.0 to 3.6 μM) and inactive *versus* Gram negative strain except for *Moraxella catarrhalis* (IC_90_ 1.3 μM).^85^ The EtOAc extract of the Hawaiian isolate of *Phoma* sp. MYC-1734 yielded phomalevone B (130) that was separated using Sephadex LH-20 and HPLC and characterized by NMR, MS, and ECD analyses. Compound 130 with bis-benzophenone skeleton displayed antimicrobial potential *versus B. subtilis, S. aureus, C. albicans*, and *E. coli* at 100 μg per disk (IZDs ranged from 18–38 mm) in the agar disk diffusion assay (Conc. 100 μg per disk).^86^

### 3.3. Cytotoxicity activity

Cancer is one of the most leading causes of death world-wide. In 2018, 9.6 million deaths because of cancer were stated according to WHO (World Health Organization). All over the world, it is estimated that ≈18.1 million cancer patients are present and this is expected to increase to 24 million in the coming decades.^91^ Since the 1980s, cancer mortality has steadily increased because of various factors, including environmental conditions and dietary habits.^92^ The most frequent and efficient cancer treatment strategies include chemotherapy and radiation therapy and surgical operation for early-stage cancers.^93^ Unfortunately, within a few years after cancer treatment, recurrence is observed with a rate of up to 70% according to cancer stages and types.^94^ Actually, the management of recurrent cancer could be hard because of their increased aggression and metastatic capacity caused by their impedance to formerly utilized drugs.^95^

BPs were tested for their cytotoxic capacity against various cancer cell lines using MTT or SRB assay. These reports were highlighted below (Table 3).

**Table 3:** Cytotoxic activity of the reported fungal benzophenones

Compd no	Cell line^a^	Biological results (IC_50_, μM)		Ref.
		Compound	Positive control	
10	HepG2^a^	63.5	5-Fu 19.2	36
25	HepG2^a^	60.2	5-Fu 19.2	36
68	A549^a^	1.8	Adriamycin 0.49	57
	HeLa^a^	2.0	Adriamycin 0.11	57
	HepG2^a^	2.2	Adriamycin 0.79	57
	MCF-7^a^	2.0	Adriamycin 0.43	57
	Vero^a^	1.5	—	57
80	A549^a^	3.7	Adriamycin 0.49	57
	HeLa^a^	5.1	Adriamycin 0.11	57
	HepG2^a^	4.5	Adriamycin 0.79	57
	MCF-7^a^	10.4	Adriamycin 0.43	57
	Vero^a^	1.7	—	57
	PANC-1^a^	14.0	5-Fu 15.0	57
81	PANC-1^a^	26.0	5-Fu 15.0	69
83	PANC-1^a^	7.6	5-Fu 15.0	69
84	PANC-1^a^	7.2	5-Fu 15.0	69
	PANC-1^a^	4.8	Cisplatin 4.0	69
	A549^a^	7.8	Cisplatin 12.0	69
	HCT-116^a^	5.5	Cisplatin 13.0	69
	MCF-7^a^	7.5	Cisplatin 22.0	69
	DU-145^a^	14.0	Cisplatin 1.9	69
	HepG2^a^	23.0	Cisplatin 10.0	69
	A549^a^	3.8	Adriamycin 0.49	57
	HeLa^a^	3.3	Adriamycin 0.11	57
	HepG2^a^	5.4	Adriamycin 0.79	57
	MCF-7^a^	5.1	Adriamycin 0.43	57
	Vero^a^	2.1	—	57
85	PANC-1^a^	14.0	5-Fu 15.0	69
	PANC-1^a^	13.0	Cisplatin 4.0	69
	A549^a^	14.0	Cisplatin 12.0	69
	HCT-116^a^	10.0	Cisplatin 13.0	69
	MCF-7^a^	11.0	Cisplatin 22.0	69
	DU-145^a^	21.0	Cisplatin 1.9	69
	HepG2^a^	37.0	Cisplatin 10.0	69
	A549^a^	5.7	Adriamycin 0.49	57
	HeLa^a^	4.7	Adriamycin 0.11	57
	HepG2^a^	5.5	Adriamycin 0.79	57
	MCF-7^a^	9.7	Adriamycin 0.43	57
	Vero^a^	3.2	—	57
86/87 mixture	PANC-1^a^	14.0	5-Fu 15.0	69
	PANC-1^a^	22.0	Cisplatin 4.0	69
	A549^a^	18.0	Cisplatin 12.0	69
	HCT-116^a^	19.0	Cisplatin 13.0	69
	MCF-7^a^	22.0	Cisplatin 22.0	69
	DU-145^a^	28.0	Cisplatin 1.9	69
99	BGC-823^a^	6.8	Adriamycin 1.5	76
	H460^a^	23.6	Adriamycin 1.0	76
	PC-3^a^	28.1	Adriamycin 1.8	76
	SMMC-7721^a^	7.9	Adriamycin 2.2	76
127	HepG2^a^	5.2	5-Fu 19.2	36
129	CHO^a^	9	—	85
	HepG2^a^	25	—	85
	MRC5^a^	13	—	85
	HEK293^a^	20	—	85
146	RKO^a^	0.8	Etoposide 3.3	88
	SNU638^a^	4.8	Etoposide 0.3	88
	SK-HEP-1^a^	2.9	Etoposide 0.4	88
	MAD-MB-231^a^	7.0	Etoposide 10.1	88
147	RKO^a^	1.1	Etoposide 3.3	88
	SNU638^a^	8.0	Etoposide 0.3	88
	SK-HEP-1^a^	3.5	Etoposide 0.4	88
	MAD-MB-231^a^	9.7	Etoposide 10.1	88
	PKO^b^	0.93	Etoposide 1.96	95
	HCT-116^b^	3.12	Etoposide 0.66	95
	SW480^b^	2.37	Etoposide 1.11	95
	Ls174T^b^	6.36	Etoposide 0.48	95
	CCD-841CoN^b^	47.18	Etoposide 8.71	95
	CCD-18Co^b^	39.11	Etoposide 18.42	95

a MTT assay.

b SRB assay; 5-Fu: 5-fluorouracil.

Ming *et al.* purified a new benzophenone, digriseophene A (2) and formerly reported 54–56 from *Corydlis tomentella*-derived *Penicillium* sp. ct28 that were established by HRESIMS and NMR analyses. The potential cytotoxic activity of these compounds was evaluated *versus* A549, Eca109, HepG2, and MDA-MB-231 cell lines using MTT assay. Compounds 2 and 56 exhibited inhibitory potential against the proliferation of A549, Eca109, HepG2, and MDA-MB-231 cell lines (IC_50_s ranged from 22.17 to 49.43 μM), comparing to vincristine (IC_50_ 0.35–1.47 μM)^60^ (Table 3).

Cytotoxic evaluation of 7 and 80*versus* HepG2, U2OS, and MCF-7 cell lines in the MTT revealed that only 7 had cytotoxic influence *versus* MCF-7 and U2OS cells (IC_50_s 16.8 and 11.6 μM, respectively).^26^ Additionally, in the MTT assay *versus* A549, HeLa and HepG2 cell lines of 8, 17, and 18, only 18 had cytotoxic potential *versus* A549 cell lines (IC_50_ 15.7 μg mL^−1^).^31^

Cytosporaphenone A (43), a new polyhydric benzophenone was isolated from Morinda officinalis-accompanied *Cytospora rhizophorae* A761 by SiO_2_/RP-18/Sephadex LH-20 CC and characterized by spectroscopic and Xray analyses. It revealed weak growth inhibition potential against MCF-7 and HepG-2 (IC_50_ 70.0 and 60.0 μM, respectively) in the SRB method.^52^

**Fig. 6:**
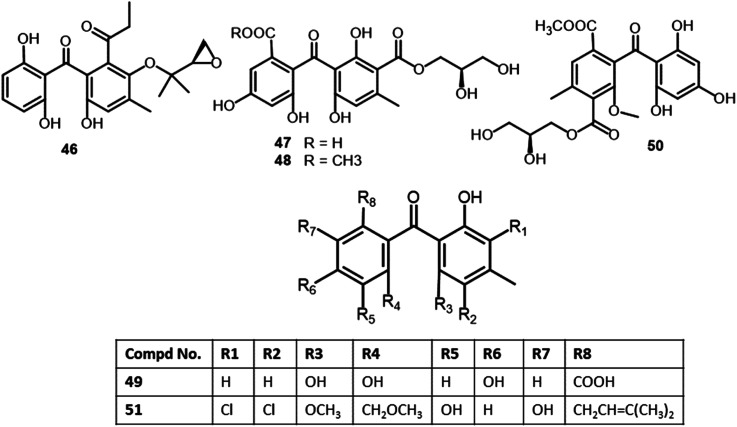
Structures of benzophenones 46–51.

**Scheme 3:**
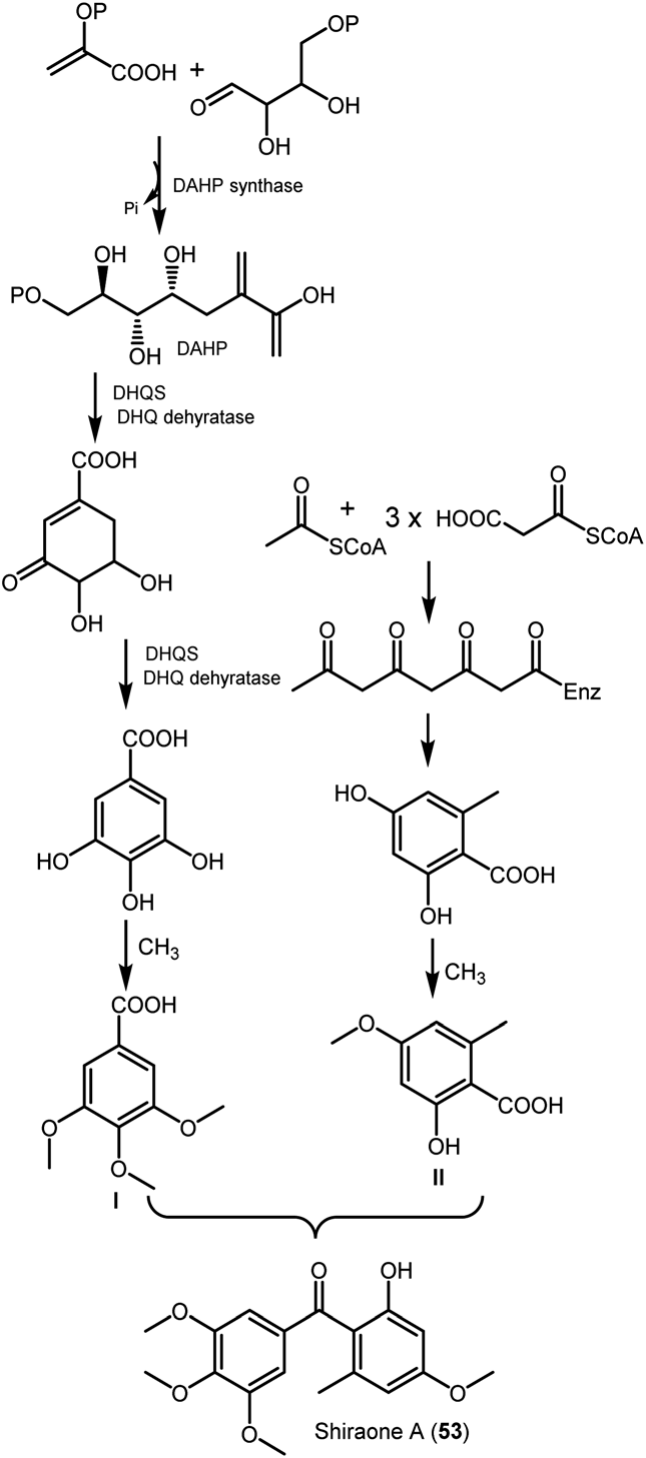
Biosynthetic pathway of shiraone A (53).^59^ DAHP: 3-deoxy-*d*-arabino-heptulosonate 7-phosphate; DHQS, 3-dehydroquinate synthase.

Chen *et al.* purified shiraone A (53), a new benzophenone derivative from the cultures of *Shiraia* sp. BYJB-1isolated from *Selaginella delicatula* leaves that was characterized by NMR, HRMS, and comparing with literature (Fig. 6). It had no cytotoxic effectiveness *versus* SMMC7721 cell line.^59^ This compound was proposed to be biosynthesized from 3,4,5-trimethoxybenzoic acid (I) and 2-hydroxy-4-methoxy-6-methylbenzoic acid (II), that were formed by shikimic acid and acetate-malonate pathways, respectively^59^ (Scheme 3).

**Fig. 7:**
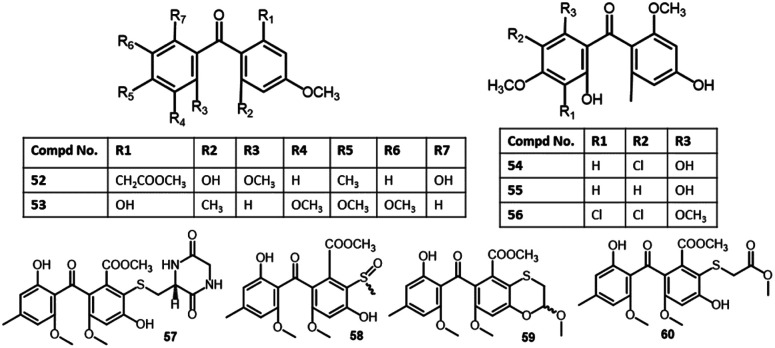
Structures of benzophenones 52–60.

Xu *et al.* reported the separation of five new benzophenone derivatives: tenellones D–H (73–77), sharing a rare aldehyde at C-2 and isoprenyl at C-6, together with the known metabolite 69 from marine sediment-derived *Phomopsis lithocarpus* FS508 using SiO_2_/Sephadex LH-20/semipreparative HPLC, which were assigned by spectroscopic and Xray analyses. Their cytotoxic activities *versus* SF-268, HepG-2, MCF-7, and A549 cell lines revealed the moderate effectiveness of 77*versus* HepG-2 and A549 cell lines (IC_50_s 16.0 and 17.6 μM, respectively). Whilst other compounds had no cytotoxic capacity even at Conc. 50 μM (Fig. 7).^65^

**Fig. 8:**
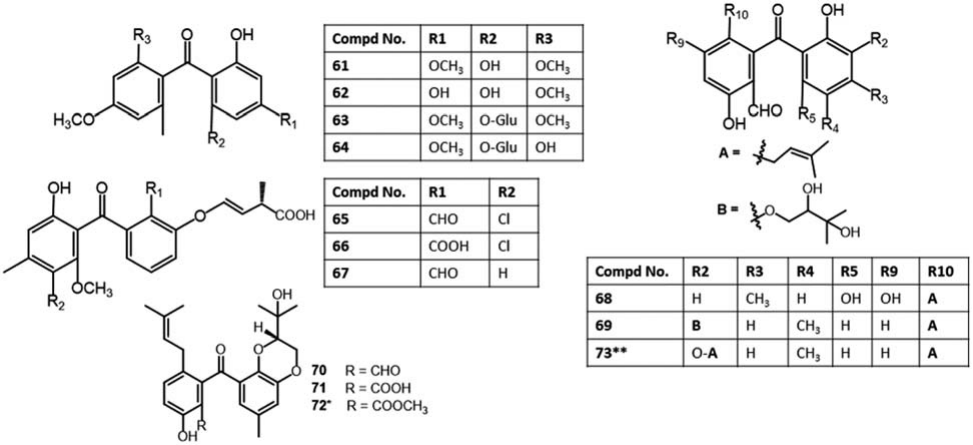
Structures of benzophenones 61–73. *, ** Same nomenclature but different structures.

It was noted that metabolites with an isoprenyl group in ring A had no activity (*e.g.*, 77*vs.*73–76 and 69)^65^ (Fig. 8). Additionally, new benzophenone analogues: 78, 79, 103, and 104 were characterized from the same fungus by Liu *et al.* utilizing NMR, ECD, and Xray analyses. Their potential anticancer activities *versus* SF-268, MCF-7, HepG-2, and A549 cell lines were evaluated using the SRB method. Compound 103 demonstrated moderate inhibition potential *versus* SF-268 cell line (IC_50_ 11.36 μM), comparing to cisplatin (IC_50_ 3.25 μM), while, 78, 79, and 104 had weak effectiveness (IC_50_s 29.49–44.48 μM).^66^ On the other hand, 129 showed cytotoxicity towards CHO, HepG2, MRC5, and HEK293 with IC_50_ 9.0–25.0 μM in the MTT assay.^85^

**Fig. 9:**
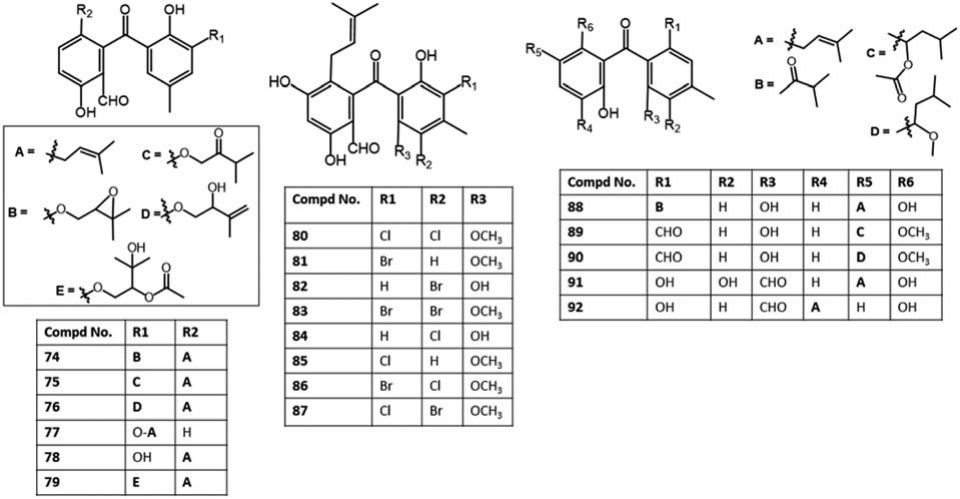
Structures of benzophenones 74–92.

New halogenated benzophenone derivatives: pestalones B–H (81–87), in addition to 80 and 99 were obtained from the EtOAc extract of *Pestalotiopsis neglecta* that was cultured in fermentation media supplemented with halide salts using SiO_2_ CC and RP-HPLC and defined by spectroscopic and Xray analyses (Fig. 9). Compounds 82 and 84 displayed the most powerful anti-proliferation potential *versus* PANC-1 cells (IC_50_s 7.6 and 7.2 μM, respectively), comparing to 5-Fu (IC_50_ 15.0 μM), while 85, 80, and 86/87 mixture had less potent effectiveness (IC_50_ 14.0 μM) than 82 and 84 but better than 81 (IC_50_ 26.0 μM) in the MTT assay. It was indicated that a second halogen atom and/or a methoxy in ring B substitution had no effect on the potency of these metabolites. In addition, 82 and 84 significantly repressed the PANC-1 cells' colony formation in the colony formation assay that supported their anti-proliferation ability of PANC-1 cells *via* boosting the caspase-3 and PARP's cleavage resulting in PANC-1 apoptosis.^69^ They possible induced their effect through prohibition of ERK/MEK pathway.^69^

In the cytotoxicity assay, 68, 80, 84, and 85 with a C-14 aldehyde group exhibited cytotoxic effectiveness (IC_50_ < 10.0 μM) *versus* A549, HeLa, HepG2, MCF-7, and Vero in the MTT assay, whereas 51, 97, 98, and 99 with oxygenated CH_2_-14 had no (IC_50_ > 50.0 μM) or weak cytotoxic potential (IC_50_: 23.2–35.8 μM) towards the tested cells, revealing the substantial role of C-14 aldehyde in cytotoxic effect of pestalones and related congeners. Whilst chlorination slightly decreased the activity.^57^

The red alga *Grateloupia turuturu*-derived *Penicillium chrysogenum* AD-1540 yielded two new benzophenone derivatives 95 and 96. Their structures and configuration were characterized relying on spectroscopic, coupling constants, and TDDFT calculations of ECD spectra. These metabolites are structural related to xanthones, while they featured an uncommon fused dihydropyran ring and an opened ring C. Both compounds revealed moderate to weak cytotoxic potential (IC_50_s 20.4–46.7 μM) *versus* BT-549, A549, HeLa, MCF-7, HepG2, and THP-1 cell lines in the CCK-8 method compared to epirubicin (IC_50_s 2.9 to 7.2 μM).^74^

**Fig. 10:**
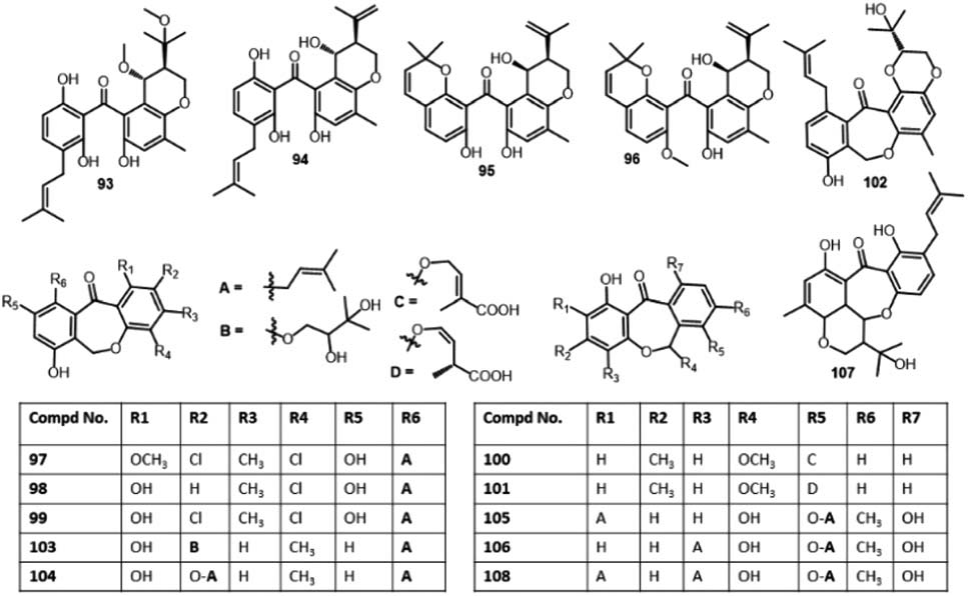
Structures of benzophenones 93–108.

In 2017, Lei *et al.* also reported the separation of 99 from a culture of *Phakellia fusca*-associated *Pestalotiopsis heterocornis* that was assessed for cytotoxic potential *versus* BGC-823, H460, PC-3, and SMMC-7721 in the MTT assay (Fig. 10). This compound displayed marked activity *versus* BGC-823 and SMMC-7721 (IC_50_s 6.8 and 7.9 μM, respectively) compared to adriamycin (IC_50_s 1.5 and 2.2, respectively), whereas it was moderately active *versus* PC-3 and H460 (IC_50_s 28.1 and 23.6 μM, respectively).^76^

**Fig. 11:**
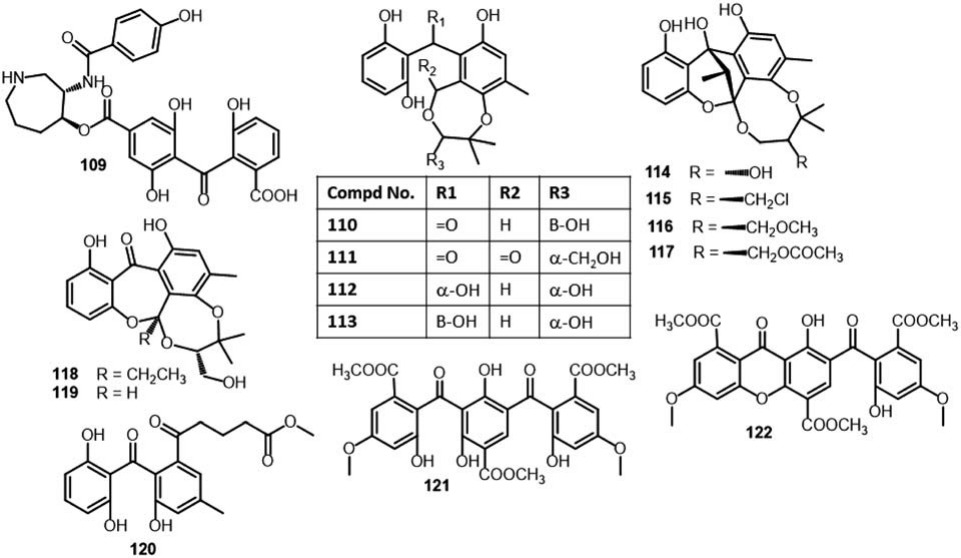
Structures of benzophenones 109–122.

**Fig. 12:**
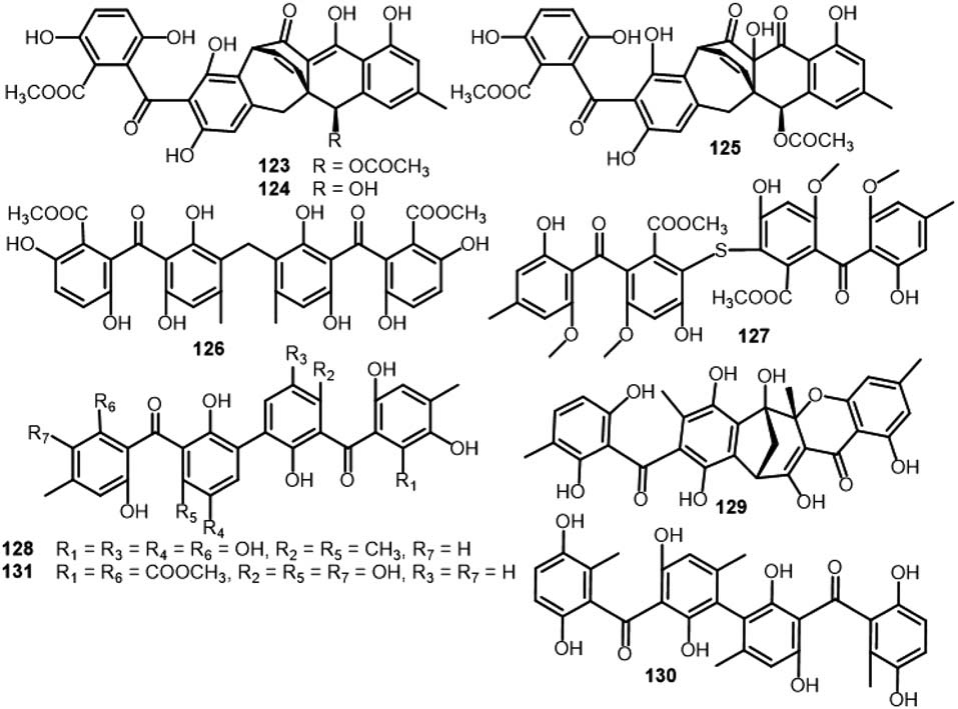
Structures of benzophenones 123–131.

The cytotoxicity investigation of 114–117*versus* HepG-2, H460, MCF-7, and SF-268 cell lines in the SRB method revealed the weak potential of 115 and 117 (IC_50_ ranged from 29.4 to 68.6 μM) *versus* these cell lines^82^ (Fig. 11 and 12). Compounds 10, 25, and 127 were assessed for their cytotoxic potential *versus* HepG2 using the MTT assay. Among them, 127 was the most active (IC_50_ 5.2 μM) than the its related monomers 10 and 25 (IC_50_s 63.5 and 60.2 μM, respectively) in comparison to 5-Fu (IC_50_ 19.2 μM).^36^

**Fig. 13:**
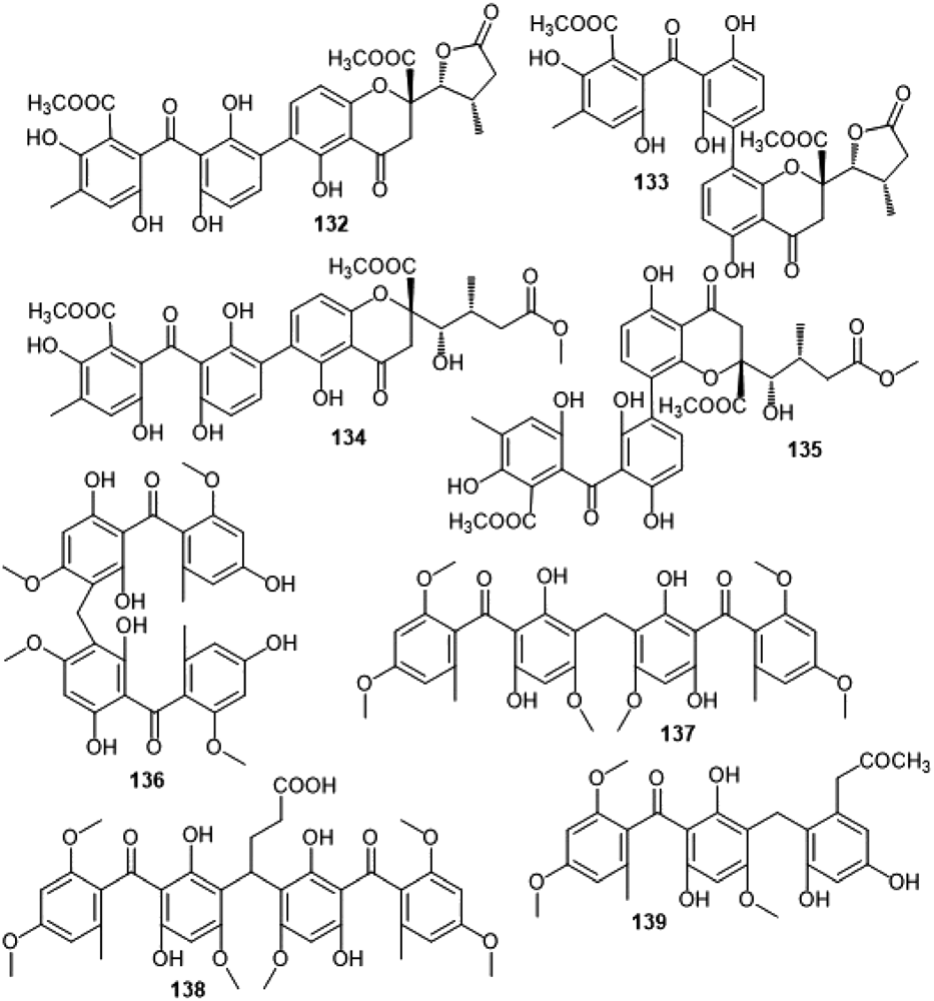
Structures of benzophenones 132–139.

From the unidentified fungus MSX 17022 belonging to Hypocreales, 1, 123, and 125 were separated (Fig. 12). In the SRB assay, 123 possessed cytotoxic effectiveness *versus* MCF-7, H460, and SF268 (IC_50_s 18.1, 13.6, and 21.4 μM, respectively), however, 125 had noticeable activity *versus* H460 and SF268 (IC_50_s 20.6 and 21.0 μM, respectively).^19^ In the brine shrimp lethality, 28 exhibited lethality potential (LD_50_ 25.3 μM), compared to colchicine (LD_50_ 1.22 μM). Also, 10 and 127 reported from *Solanum insanum*-associated *Aspergillus fumigatus* displayed brine shrimp toxicity (IC_50_ 74.2 μM) (Fig. 13).^37^

In 2017, Liao *et al.* reported the purification of novel diastereomeric lipo-peptidyl benzophenones: asperphenins A (146) and B (147) from the MeOH extract of marine-derived *Aspergillus* sp. using RP-18 CC and HPLC, which were characterized based on spectroscopic, CD, and ECD analyses, as well as Mosher's method. These compounds are C-17 epimers, having *R* and S configuration, respectively and their structures involve trihydroxybenzophenone, 3-hydroxydodecanoic acid, and tripeptide moieties. Both 146 and 147 exhibited significant antiproliferative activity *versus* RKO, SNU638, SK-HEP-1, and MDA-MB-231 cell lines (IC_50_s ranged from 0.8 to 9.7 μM) in the MTT assay. It is worth that RKO cells were the most sensitive cell lines towards 146 and 147 (IC_50_s 0.8 and 1.1 μM, respectively), compared to etoposide (IC_50_ 3.3 μM).^88^ In 2020, Bae *et al.* also reported the antitumor potential of 146 and 147*versus* SK-HEP-1, RKO, MAD-MB-231, and SNU638 cell lines (IC_50_s ranged 0.84–6.48 μM for 146 and 1.26–9.43 μM for 147). Further, studying the antiproliferative mechanism of 146 on RKO cells revealed that 146 suppressed RKO growth *via* arresting G2/M cell cycle through prohibiting microtubule polymerization with subsequent apoptosis. It also induced reactive oxygen species and repressed the tumor growth in a colon cancer xenograft model without any toxicity. Interestingly, it possessed synergistic influence with irinotecan (topoisomerase I inhibitor), however, it had antagonistic influence with paclitaxel that indirectly supported their opposite molecular mechanisms. It was found that the aryl ketone moiety is accountable for 146's activity.^96^ Therefore, 146 could be new lead metabolite for finding out chemotherapeutic agents with antimitotic capacity.

In the same aspect, Byun *et al.* demonstrated that 147 possessed potent cytotoxic potential *versus* human CRC (colorectal cancer) cell lines: HCT-116, RKO, Ls174T, and SW480 (IC_50_s ranged from 0.93 to 47.18 μM) compared to etoposide in the SRB assay. Compound 147 was found to induce cell cycle arrest at G2/M phase and with subsequent apoptotic cell death, also, it suppressed tumor growth in a xenograft model.^95^ Its G2/M phase arrest influence was accompanied with the check-point proteins (Cdc25c and Chk1/2) regulation, whereas its apoptosis potential was linked to survivin down-regulation and cleaved caspases and p53 upregulation. Further, it boosted the repression of HCT-116 cells invasion and migration through GAPDH (glyceraldehyde-3-phosphate dehydrogenase) downregulation. Also, it upregulated E-cadherin and down-regulated Snail and N-cadherin, confirming its antimetastatic effectiveness. Hence, its antimetastatic and antitumor potential was determined to be due to modulating GAPDH-induced EMT processes. This highlighted the potential of 147 as promising candidate for metastatic CRC treatment.^95^

### 3.4. Antioxidant activity

Some of the reported BPs possessed potent antioxidant potential than positive controls. Herein, the reported studies on the antioxidant activity were discussed and the results were listed in Table 4.

**Table 4:** Antioxidant activity of the reported fungal benzophenones^a^

Compd no.	Assay/cell line	Biological results		Ref.
		Compound	Positive control	
8	DPPH	18.9 μM*	Ascorbic acid 11.86 μM*	33
	CCK-8/PC-12	37.38**	Vitamin E 57.68**	33
10	CCK-8/PC-12	51.66**	Vitamin E 57.68**	33
12	DPPH	2.3 μg mL^−1^*	Trolox 5.4 μg mL−1*	30
13	DPPH	5.4 μg mL^−1^*	Trolox 5.4 μg mL−1*	30
37	ABTS	0.69 μg mL^−1^****	Ascorbic acid 3.01 μg mL−1****	48
38	DPPH	1.33 μg mL^−1^*	BHT 16.27 μg mL−1*	48
	ABTS	0.58 μg mL^−1^*	Ascorbic acid 3.01 μg mL−1*	48
46	DPPH	13.07 μM***	Ascorbic acid 25.53 μM***	55
49	DPPH	1.7 μg mL^−1^*	Trolox 5.4 μg mL−1*	30
52	DPPH	28.62 μM*	Ascorbic acid 25.13 μM*	58
57	CCK-8/PC-12	54.22**	Vitamin E 57.68**	33
58	CCK-8/PC-12	62.40**	Vitamin E 57.68**	33
59	CCK-8/PC-12	63.24**	Vitamin E 57.68**	33
60	CCK-8/PC-12	49.11**	Vitamin E 57.68**	33
120	DPPH	9.5 μM***	Ascorbic acid 21.9 μM***	83

a *IC_50_; ** % viability; ***: EC_50_.

In the DPPH assay, 37 and 38 exhibited stronger scavenging potential for DPPH and ABTS radicals (IC_50_ 1.26 and 1.33 μg mL^−1^, respectively for DPPH and 0.69 and 0.58 μg mL^−1^, respectively for ABTS) compared to BHT (butylated hydroxytoluene, IC_50_ 16.27 μg mL^−1^ for DPPH) and ascorbic acid (IC_50_ 3.01 μg mL^−1^ for ABTS).^48^ A novel derivative, rhizophol A (46) was isolated from the endophytic fungus *Cytospora rhizophorae* A761 and characterized by NMR and Xray, as well as quantum energy calculation. This compound featured unrivalled substituted benzophenone framework, having propionyl and epoxy isopentyl moieties. Compound 46 revealed marked DPPH scavenging capacity (EC_50_ 13.07 μM), which was powerful than ascorbic acid (EC_50_ 25.53 μM) in the DPPH assay, suggesting its potential as prominent lead compound for developing novel antioxidant drug.^55^

*Xestospongia testudinaria*-associated *Aspergillus europaeus* WZXY-SX-4-1 biosynthesized new derivatives: eurobenzophenones A–C (47–49), alongside 8 and 12–15 that were isolated using RP-18 CC and RP-HPLC, in addition their structures were established by spectroscopic analyses, as well as Snatzke method for configuration assignment. Compounds 47 and 48 possess a C15 ester with 2′*R*-configured glycerol moiety, where 48 is a methyl ester of 47. Benzophenones 12, 13, and 49 revealed powerful DPPH radical scavenging potential (IC_50_s 2.3, 5.4, and 1.7 μg mL^−1^ respectively), while other metabolites had moderate efficacy (IC_50_ s ranged 11.6–25.3 μg mL^−1^), compared with trolox (IC_50_ 5.4 μg mL^−1^).^30^

The new metabolite, 52 obtained from *Aspergillus fumigatus* SZW01 had significant free radical scavenging capability. In the ABTS assay, 52 possessed stronger potential than ascorbic acid (IC_50_ 12.5 μM), however, it had relatively weak potential (IC_50_ 28.62 μM) in the DPPH assay compared to ascorbic acid (IC_50_ 25.13 μM).^58^ Cave soil-derived *Aspergillus fumigatus* GZWMJZ152 yielded new sulphur-having benzophenones: 57–60, in addition to 8 and 10 that were separated utilizing SiO_2_/Sephadex LH-20/RP-18 CC and preparative TLC. Their structures and absolute configurations were proved by spectroscopic, X-ray, and ECD analyses. Compound 57 represents an uncommon hybrid of diketopiperazine-benzophenone *via* a thioether linkage. Compound 57 with 6′*R*-configuration involves cyclo-Gly-Cys diketopiperazine that is *S*-linked to monomethylsulochrin framework (10). Both 58 and 59 were initially separated as racemic mixtures that were then purified as the enantiomerically pure (+)-58, (−)-58, (+)-59, and (−)-59, respectively. Compound 58 was assigned as *R*-(+)- and *S*-(−)-2-methylsulfinyl monomethylsulochrin that have rare methyl sulfinyl group, while 59 featured 2-methoxy-1,4-oxathiane that was linked into the C-2/C-3 bond of sulochrin nucleus. Besides, 60 has a methyl mercapto-acetate moiety connected *via* a thioether to the C-2 of monomethylsulochrin nucleus. These metabolites were investigated for antioxidant potential by assessing DPPH scavenging potential and ORAC index as well as protective potential *versus* H_2_O_2_-produced oxidative damage on PC12 cells. The results revealed that 8 scavenged DPPH radicals (IC_50_ 18.90 μM), compared to vitamin C (IC_50_ 11.86 μM), while 10, 57, (±)-58, (+)-58, (−)-58, (±)-59, (+)-59, and (−)-59 exhibited potent antioxidant capacities with ORAC ranging from 0.02 to 6.14 μM TE μM^−1^. Furthermore, compounds 8, 57, (±)-58, (±)-59, and 60 revealed protection efficacy on H_2_O_2_-induced oxidative injury on PC12 cells (% viability 51.66, 54.22, 62.4, and 63.24, respectively) in the CCK-8 assay compared to vitamin E (% viability 57.68).^33^ Recently, cytorhizophin J (120) was obtained from the EtOAc extract of Cytospora heveae NSHSJ-2 isolated from the fresh stem of Sonneratia caseolaris by SiO_2_, Sephadex LH-20 CC, and HPLC. This compound was similar to 45 with C-13 5-methoxy-5-oxopentanoyl moiety instead of propionyl group at the C-13 in 45. It (EC_50_ 9.5 μM) showed marked antioxidant potential compared to ascorbic acid (EC_50_ 21.9 μM) in the DPPH assay.^83^

### 3.5. Immune-suppressive activity

Most of the immunological disorders are resulted from immune cells' abnormally low or over activity. In immune-system overactivity, the body damages and attacks its own tissues referring to an acquired immune system reaction. Immune-suppressants are utilized to control autoimmune disorders and improved allograft survival, however, they possess deleterious side effects.^97^

From the EtOAc extract of *Penicillium* sp. ZJ-SY2 isolated from *Sonneratia apetala* leaves, two new benzophenone derivatives; peniphenone (39) and methyl peniphenone (40) were separated using SiO_2_/Sephadex LH-20/RP-HPLC. Their immunosuppressive potential *versus* Con A-caused T cell and LPS-induced B cell proliferations of mouse splenic lymphocytes in the MTT method was evaluated. Compound 39 displayed potent immunosuppressive effectiveness (IC_50_s 9.3 and 8.1 μg mL^−1^, respectively) *versus* LPS- and Con A–induced proliferations of mouse splenic lymphocytes compared to azathioprine (IC_50_ 2.7 μg mL^−1^), while 40 had weak influence (IC_50_s 23.7 and 17.5 μg mL^−1^, respectively) (Table 5). It was found that C-1 carboxylic acid group boosted the activity, compared to 40 bearing a methyl ester group.^49^

**Table 5:** Other activities of the reported fungal benzophenones

Compound name	Assay, organism, or cell line	Biological results (IC_50_)		Ref.
		Compound	Positive control	
**Anti-inflammatory**				
3-de-*O*-Methylsulochrin (12)	LPS/Spectrophotometric	71.0%^a^	MG132 88.9%^a^	30
Dipleosporone A (137)	LPS/Spectrophotometric	8.8 μM	Dexamethasone 22.2 μM	63
Dipleosporone B (138)	LPS/Spectrophotometric	15.6 μM	Dexamethasone 22.2 μM	63
Dipleosporone C (139)	LPS/Spectrophotometric	18.1 μM	Dexamethasone 22.2 μM	63

**Antimalarial**				
Orbiocrellone B (132)	GFP/*P. falciparum* K1	5.7 μM	Dihydroartemisinin 0.0025 μM	53
Orbiocrellone C (133)	GFP/*P. falciparum* K1	5.6 μM	Dihydroartemisinin 0.0025 μM	53
Orbiocrellone D (134)	GFP/*P. falciparum* K1	14.0 μM	Dihydroartemisinin 0.0025 μM	53
Ent-secalonic acid I (144)	GFP/*P. falciparum* K1	5.5 μM	Dihydroartemisinin 0.0025 μM	53

**SOAT inhibitory**				
FD549 (88)	SOAT1, African green monkey (CHO)/Cell based	9.9 μM	—	70
	SOAT2, African green monkey (CHO)/Cell based	0.91 μM	—	70
	SOAT1, Human/Cell based	5.2 μM	—	70
	SOAT2, Human/Cell based	0.68 μM	—	70
Celludinone B (143)	SOAT1, African green monkey (CHO)/Cell based	2.8 μM	—	70
	SOAT2, African green monkey (CHO)/Cell based	0.15 μM	—	70
	SOAT1, Human/Cell based	2.9 μM		70
	SOAT2, Human/Cell based	0.069 μM		70

**α-Glucosidase inhibitory**				
3-de-*O*-Methylsulochrin (12)	Colorimetric	0.199 μM	Quercetin 0.015 μM	38
			Acarbose 0.685 μM	

**Immunosuppressive**				
Peniphenone (39)	Mouse splenic lymphocytes/Con-A	8.1 μg mL^−1^	Azathioprine 2.7 μg mL^−1^	49
	Mouse splenic lymphocytes/LPS	9.3 μg mL^−1^	Azathioprine 2.7 μg mL^−1^	49

**Anti-toxoplasmosis**				
Tenellone A (69)	*Eimeria tenella* (EtPKG)/radiometrically	12.6 μM	Synthetic refence compound <0.001 μM	64
Tenellone B (70)	*Eimeria tenella* (EtPKG)/radiometrically	8.7 μM	Synthetic refence compound <0.001 μM	64

**Anti-coccidiosis**				
Tenellone A (69)	*Toxoplasma gondii* (TgWC)/β-galactosidase, colorimetrically	1.8 μM	Synthetic refence compound 210.0 μM	64

### 3.6. Anticoccidial and anti-malarial activities

*Eimeria* spp. causes coccidiosis, which is a significant parasitic disease affects chickens, resulting in serious economic losses through mortality and morbidity. The anticoccidial agents such as polyether ionophore are successfully utilized in poultry industry. Unfortunately, resistance has been observed to the existing anti-coccidiosis agents, therefore, search for new therapeutic agents for coccidiosis control are needed.^98^

Bioassay-guided fractionation of *Diaporthe* sp. associated with *Aeonium cuneatum* stems resulted in the purification of 69 and 70, two new highly substituted benzophenones from the methyl ethyl ketone extract using Sephadex LH-20 and HPLC, which were determined by spectroscopic and Xray analyses. They featured trioxygenated isopentane and 1,4-dioxane moieties, respectively. Their *Eimeria tenella* PKG (cGMP-dependent-protein kinase) and *Toxoplasma gondii* whole cell (TgWC) inhibition capability was estimated using radiometric and β-galactosidase whole cell reporter assays, respectively. Compound 69 prohibited EtPKG (IC_50_ 12.6 μM) and had notable TgWC inhibitory potential (IC_50_ 1.8 μM) compared to a synthetic reference (IC_50_ < 0.001 and 210.0 μM, respectively), while 70 demonstrated potential on EtPKG (IC_50_ 8.7 μM). Unfortunately, neither 69 nor 70 displayed anticoccidial potential on *Eimeria*-affected chickens (dose 100 ppm).^64^

Investigation of the insect-associated *Orbiocrella petchii* BCC 51377 EtOAc extract using RP-18/SiO_2_/Sephadex LH-20 CC and RP-HPLC resulted in orbiophenone A (44, benzophenone derivative), orbiocrellone A (131, homodimer of 44), orbiocrellones B–E (132–135, chromone-benzophenone heterodimers), and ent-secalonic acid I (144, tetrahydroxanthone-benzophenone dimer) that were elucidated by spectroscopic and chemical analyses, additionally their absolute configuration was established by ECD spectra and ECD-TD-DFT calculation. Compound 131 is a C-11-C-11′ symmetric homodimer of 44. Besides, 133 is an isomer of 132, differing in the dimerization position and 144 with 5′*S*/6′*R*/10a′*S* configuration is an enantiomer secalonic acid I formerly reported from *Penicillium oxalicum*.^99^ Compounds 132, 133, 134, and 144 revealed antimalarial potential *versus Plasmodium falciparum* K1 (IC_50_s 5.7, 5.6, 14.0, and 5.5 μM, respectively) compared to dihydroartemisinin (IC_50_ 0.0025 μM) in the microculture radioisotope technique.^53^

### 3.7. Anti-inflammation activity

The new derivatives: eurobenzophenones A–C (47–49), alongside 8 and 12–15 (Conc. 10 μM) exerted inhibition potential *versus* NO production boosted LPS in the BV2 cells (% inhibition 17.4–39.4%), compared to curcumin (% inhibition 60%). Compound 8, 12, and 48 (Conc. 10 μM) remarkably declined NF-κB expression (inhibitory rates 67.2, 71.0, and 74.9%, respectively), compared to MIG132 (NF-κB inhibitor, 90% inhibitory rate, Conc. 10 μM).^30^ The significant inhibitory potential of 48 toward NO was mediated by NF-κB down-regulation.^30^

Tenellone D (72) a new derivative along with 71 were separated from *Diaporthe* sp. SYSU-HQ3 CH_2_Cl_2_ extract by different chromatographic methods. Compound 72 is related to 71 with a methyl ester moiety instead of the carboxylic acid moiety at C-2 in 71. It was proposed that methyltransferase may be accountable for the C-1 carboxyl group methylation. It is noteworthy that 71 exhibited no inhibition on NO production boosted by LPS in the RAW 264.7 cells (Conc. 100 μM), however, its C-1 methyl ester 72 possessed (IC_50_ 18.6 μM) marked inhibitory potential, comparing to indomethacin (IC_50_ 37.5 μM), suggesting esterification enhanced the activity.^14^ Whilst 123 and 125 were inactive in assays for both NF-kB inhibition and mitochondrial transmembrane potential.^19^

New dimeric benzophenones; 137–139 and benzophenone monomers; 61, 63, and 64, along with 55 and 62 were isolated from *Pleosporales* sp. YY-4 associated with *Uncaria rhynchophylla* by SiO_2_/RP-18/HPLC and assigned by HREIMS and NMR. Compounds 137–139 are the first C bridged benzophenone dimers. These metabolites were evaluated for their anti-inflammatory activity by examining their inhibition of NO production induced by LPS in the RAW 264.7 cells using CCK-8 assay. Compounds 64 and 137–139 possessed more noticeable inhibition potential *versus* LPS-caused NO production in the RAW 264.7 cells (IC_50_ ranged from 8.8 to 23.3 μM) than dexamethasone (IC_50_ 22.2 μM). The dimeric derivatives 137–139 were more potent than the monomers 61–63 and 55 that displayed moderate anti-inflammation potential (IC_50_ ranged from 35.1 to 43.3 μM).^63^

### 3.8. α-Glucosidase, proteasome, and tyrosine phosphatase inhibitory activities

α-Glucosidase catalyses the glycosidic bonds hydrolysis of nonreducing saccharide polymers to give glucose.^100^ α-Glucosidase inhibition controls the postprandial blood level due to slowing the dietary carbohydrates uptake.^101,102^ α-Glucosidase inhibitors have been assumed to be therapeutic agents for carbohydrate-related metabolic disorders such as diabetes.

The chemical investigation of the EtOAc extract of *Aspergillus flavipes* PJ03-11 resulted in separation of a new benzophenone, 11, along with 12 and 21 by repeated SiO_2_/Sephadex LH-20 CC/RP-HPLC. Compounds 12 and 21 (IC_50_s 0.199 and 0.042 μM, respectively) demonstrated stronger α-glucosidase inhibition potential than acarbose (IC_50_ 0.685 μM) and quercetin (IC_50_ 0.015 μM), while 11 (IC_50_ > 2.0 μM) had modest activity.^38^ Further, compound 52 was reported to exhibit powerful α-glucosidase inhibition than acarbose.^58^

KATP channel has a major function in the control of β-cell membrane potential. pancreatic β-cells KATP channel inhibitors help the release of insulin and are used as antidiabetics such as sulfonylureas, however, the usage of KATP channel blockers leads to a high incidence of hypoglycaemic events.^51^ It was reported that voltage-gated K channels modulation could be an alternative in antidiabetic indications. β-cell Kv2.1 (voltage-dependent K^+^) channel contributes to insulin-secreting cell repolarization and regulates pancreatic insulin secretion.^103^ The β-cell Kv2.1 currents prohibition results in prolongation of the action potentials and sustaining voltage-dependent Ca^2+^ channels opening, therefore enhanced glucose-boosted insulin release without producing risky hypoglycaemia. Therefore, the β-cell Kv2.1 channel is targeted for T2DM treatment.^51^

**Fig. 14:**
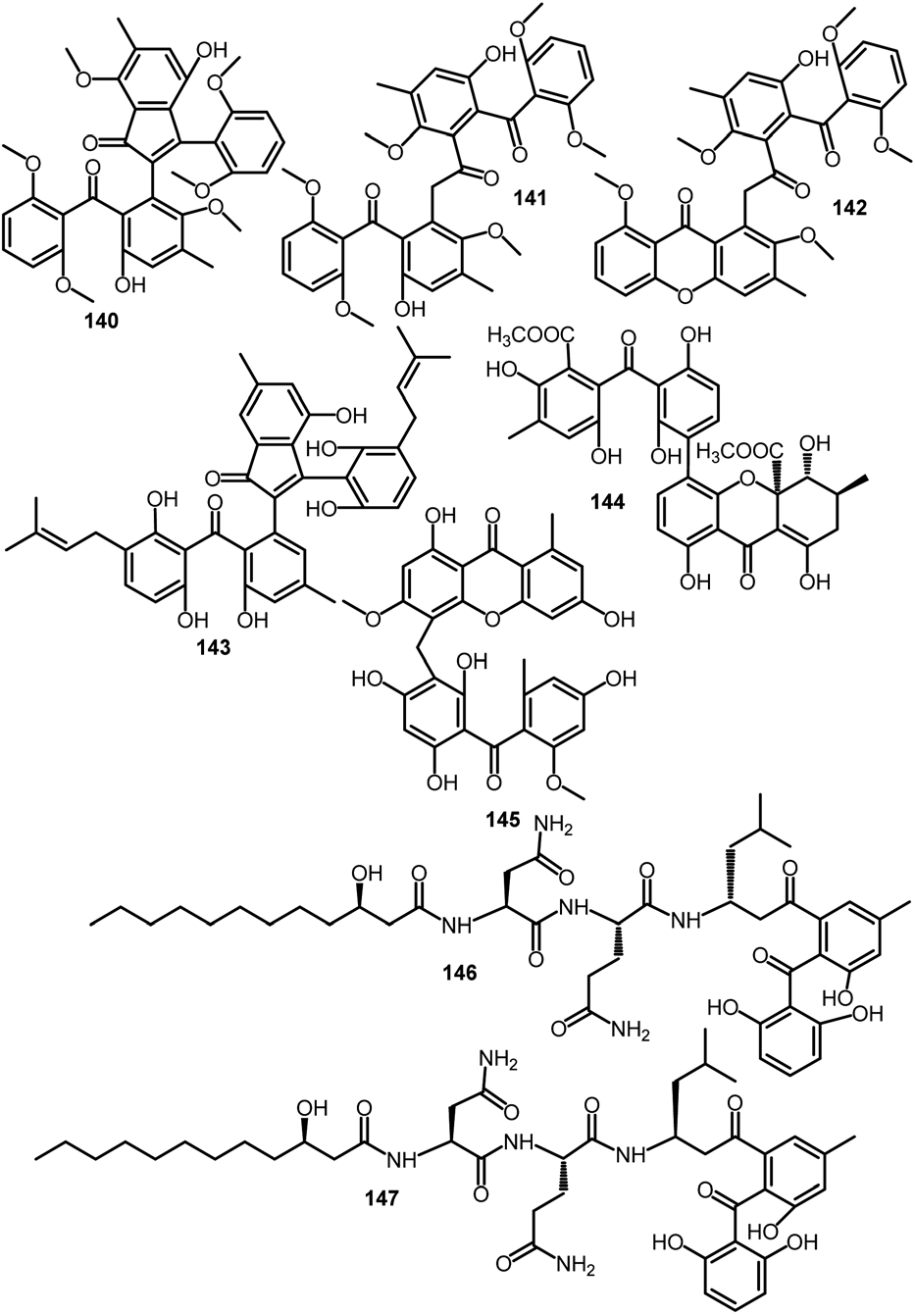
Structures of benzophenones 140–147.

Two new benzophenones, acredinones A (140) and B (141), along with 42 were separated from the marine sponge-accompanied *Acremonium* sp. EtOAc extract utilizing SiO_2_ CC and RP-HPLC (Fig. 14). Their structures were elucidated by spectroscopic data and chemical derivatization. They were assayed for inhibition of the outward K^+^ currents in INS-1. Compounds 140 and 141 revealed notable inhibitory potential on voltage-gated K^+^ channel in INS-1 cells (IC_50_s 0.59 and 1.0 μM, respectively), while 42 had no inhibitory activity.^51^ These metabolites represent the first nonpeptidic natural metabolites, possessing marked outward K+ currents prohibition in INS-1 cells.

Compounds 123 and 125 were moderately effective *versus* 20S proteasome (% inhibition 12.0 and 32.0, respectively, Conc. 5 μg mL^−1^).^19^ Also, 27 purified from ascidian-derived *Penicillium albo-biverticillium* TPU1432 culture broth using RP-18 CC and HPLC (ODS) was assessed for its inhibitory potential on PTP-1B (protein tyrosine phosphatase-1B), TCPTP (T cell PTP), and CD45 (CD45 tyrosine phosphatase) in the colorimetric assay. Compound 27 was found to have inhibitory capacity on CD45, PTP1B, and TCPTP (IC_50_s 21.0, 36.0, and 20.0 μM, respectively) compared to oleanolic acid (IC_50_ 0.8, 1.0, and 0.9 μM, respectively).^44^

### 3.9. Anti-osteoclastogenic activity

Osteoclasts overactivity results in excessive bone resorption that breaks the bone resorbing/forming balance, leading to osteopenic disorders such as Paget's disease, periodontal disease, osteoporosis, and rheumatoid arthritis.^104^ The RANK/RANKL signalling pathway activates substantial signalling molecules for osteoclast function and development.^105^ Several reports investigated the inhibitory potential of natural metabolites on RANKL-mediated osteoclast differentiation aiming at discovering new drug leads for treating osteoporosis.^106^

A new RANKL-induced osteoclast differentiation inhibitor, acredinone C (142), along with related analogs 140 and 141 were separated from *Acremonium* sp. F9A015 culture broth EtOAc extract utilizing RP-HPLC. Compound 142 incorporates xanthone and benzophenone moieties, that was established by NMR and MS analyses. These acredinones effectively prohibited the RANKL-produced formation of TRAP^+^-MNCs without any toxicity up to 10 μM. Their anti-osteoclastogenic potential was correlated with the downstream effectors' blockage *via* down-regulating of NFATc1 (nuclear factor of activated-T cells, cytoplasmic 1) expression through inhibiting signalling molecules: ERK, p38, IκBα, and AKT. Further, 140 possessed dual potential on osteo-clasto-genesis and osteo-blasto-genesis, where its osteogenic potential was due to osteoblast-specific genes up-regulation through BMP family members control and Smad signalling pathway. Additionally, 140 had marked bone-formation potential in the *in vivo* mouse model, thence, 140 could be a potential lead as an anabolic agent and/or anti-resorptive agent to prohibit and heal bone disorders.^87^

### 3.10. Antihyperlipidemic activity

Body stores excessive energy as lipid droplets in adipocytes that act as an energy reservoir. Excessive storage of lipids was found to be a cause of diverse disorders, including cardiovascular disease, T2DM, and atherosclerosis.^107^

Chemical examination *Cinachyrella* sp.-associated *Emericella variecolor* resulted in separation of a new metabolite; 19-*O*-methyl-22-methoxypre-shamixanthone (93), together with 94 using SiO_2_/RP-18/Sephadex LH-20 and semipreparative HPLC that were elucidated based on extensive spectroscopic, ECD, and Xray analysis as well as Mosher's method. These metabolites were examined for lipid-lowering potential on OA (oleic acid)-elicited lipid accumulation in the HepG2 cells by measuring Oil Red O staining. Compound 94 exerted marked lipid accumulation inhibition potential (Conc. 10 μM) comparable to that of simvastatin accompanied with potent reducing of intracellular TG (triglyceride) and TC (total cholesterol), without toxicity toward HepG2 cells up to 100 μM in the MTT assay. It mediated its lipid accumulation inhibitory potential through down-regulating the expression of the principal lipogenic transcriptional factor; SREBP-1c (sterol regulatory element-binding transcription factor 1) and its down-stream genes, including FAS (fatty acid synthase) and ACC (acetylCoA carboxylase). Thence, it lessened lipid accumulation *via* SREBP-1 pathway downregulation with no toxicity, suggesting its potential as lead compound for developing anti-hyperlipidemic agent.^73^

### 3.11. Sterol *O*-acyltransferase inhibitory activity

SOAT-2 (sterol *O*-acyltransferase-2) is belonging to the membrane-bind *O*-acyl-transferase family that adjusts the body metabolism of cholesterol.^70,108^ It is principally expressed in the small intestine and hepatocytes. It has been reported as a substantial target for treating/preventing atherosclerosis and hypercholesterolemia than SOAT1.^70^

A new indanone analog: celludinone B (143), along with 88 were purified from *Talaromyces cellulolyticus* BF-0307 culture broth by RP-18 CC and HPLC and assigned by NMR spectral data. Their SOAT (sterol *O*-acyltransferase) inhibition potential was assessed on SOAT-1(sterol *O*-acyltransferase-1) and SOAT-2 (sterol *O*-acyltransferase-2) isozymes in the cell-based assay using SOAT-1- and -2-CHO (Chinese hamster ovary) cells. Compounds 88 and 143 displayed noticeable SOAT-1 and SOAT-2 inhibitory capacity (IC_50_s 9.9 and 0.91 μM for 88 and 2.8 and 0.15 μM for 143, respectively). Interestingly, both 88 and 143 were SOAT2 selective inhibitors, suggesting that the benzophenone moiety in 88 and 143 was substantial for SOAT-2-selective inhibition (Table 5). Similar findings were noted on using human SOAT-1- and SOAT-2-expressing CHO cells without toxic effect in these cell lines even at 20 μM.^70^

### 3.12. Phytotoxic and insecticidal activities

Phytotoxic constituents act as pathogenicity or virulence factors in pathogen-host interactions and in the infectious mode.^109,110^ The separation of such metabolites assists in understanding their potential in the induction of disease symptoms and phyto-pathogenic processes that could help in assigning disease management.^109,110^ Additionally, these metabolites can be used as potential herbicides.

Rabenzophenone (2), a new hexa-substituted derivative, along with 1 were separated by SiO_2_ CC and preparative TLC from the extract of *Fimetariella rabenhorstii* obtained from *brantii brantii* (Iranian oak) infected stems. Compound 2 is related to 1, but it has an extra C-4 chlorine atom. These compounds exhibited phytotoxic potential on tomato and holm oak leaves (Conc. 1 mg mL^−1^), causing a necrosis (diameter ranged from 0.2 and 0.5 cm) in the leaf puncture bioassay, whereas 2 was the most phytotoxic one.^21^ Additionally, they were separated from the solid culture of *Alternaria sonchi (Sonchus* spp. (sowthistles) leaf pathogen). They had phytotoxic effectiveness on *Elytrigia repens* (couch-grass) and *Sonchus arvensis* (sowthistle) leaves in the punctured leaf disc assay.^110^

Compounds 8, 16, 19, and 20 were assessed for their insecticidal potential by inhibiting the growth of newly hatched *Helicoverpaarmigera* Hubner larvae. They were found to possess growth inhibition potential (IC_50_s 200, 200, 200, and 100 μg mL^−1^, respectively), compared azadirachtin (IC_50_ 50 μg mL^−1^).^32^

### 3.13. Protein kinase inhibitory activity

Chromatographic investigation of the *n*-BuOH fraction of *Verticillium balanoides* mycelia that was collected from *Pinus palustris* needle litter near Hoffman, North Carolina utilizing Sephadex LH-20 and HPLC afforded balanol (109) that was assigned by MS, Xray, and NMR data. This compound demonstrated potent PKCs (protein kinase Cs: α, β-I, β-II, γ, δ, ε, and η) inhibitory potential (IC_50_s ranged from 4–9 nM).^80^

## 4 Conclusions and future prospective

**Fig. 15:**
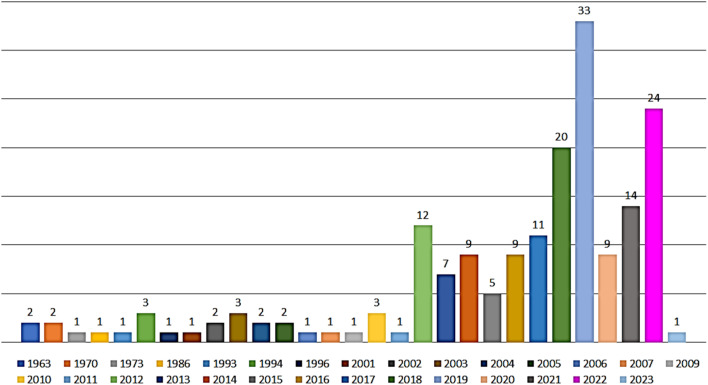
Number of benzophenones reported from fungal source per year.

It is apparent that fungi are capable of creating medicinally valuable metabolites that have been established to possess novel action mechanisms that hold great promise as prospected drug candidates. From 1963 to October 2022, 146 benzophenone derivatives were separated from fungal sources, particularly from endophytic fungi. Most of them were reported in the period from 2018 to 2022, the decrease in the number of reported metabolites in 2020 and 2021 may be due to COVID-19 pandemic (Fig. 15).

**Fig. 16:**
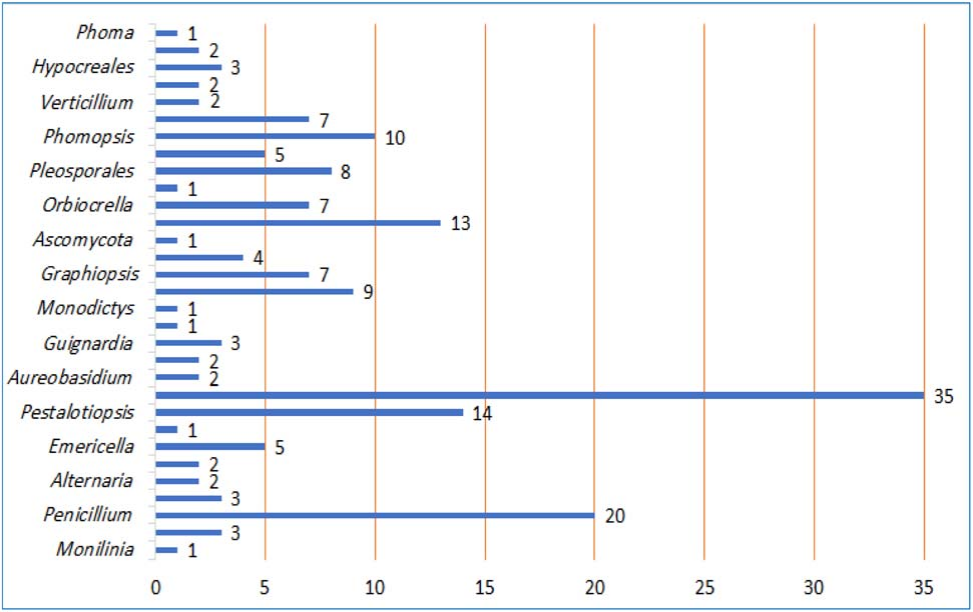
Number of benzophenones reported from various fungal genera.

**Fig. 17:**
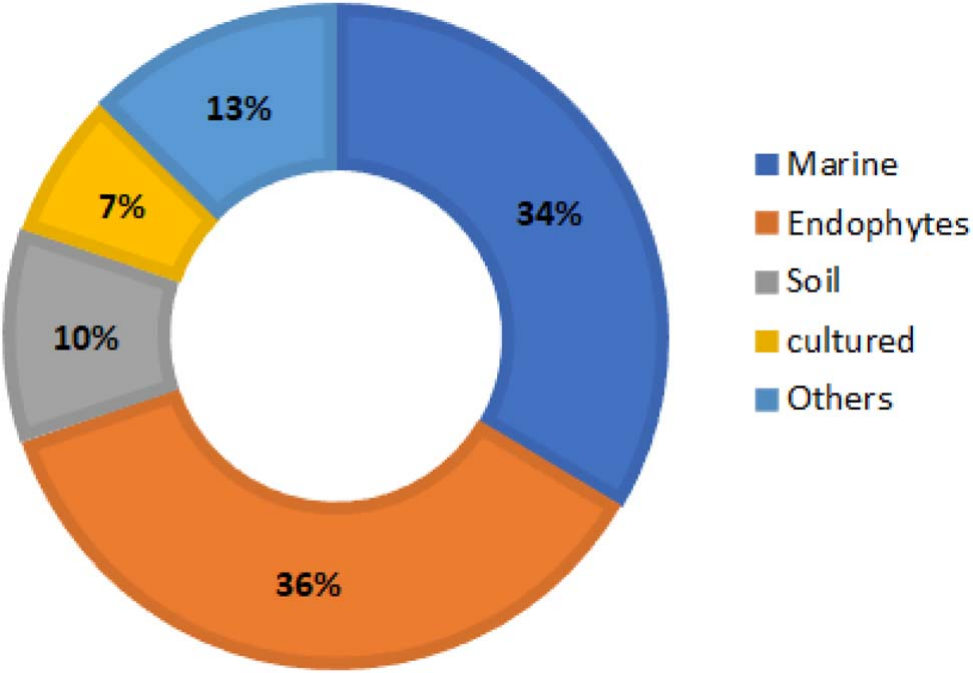
Number of benzophenones reported from fungal species derived from different sources.

These metabolites have been reported from 31 fungal genera: Monilinia, Hypocreales, Penicillium, Fimetariella, Alternaria, Daldinia, Emericella, Cercophora, Pestalotiopsis, Aspergillus, Aureobasidium, Rhizoctonia, Guignardia, Astrocystis, Monodictys, Acremonium, Graphiopsis, Talaromyces, Ascomycota, Cytospora, Orbiocrella, Shiraia, Pleosporales, Diaporthe, Phomopsis, Mericella, Verticillium, Delitschia, Hypocreales, Microsphaeropsis, and Phoma. Most of them are reported from Pestalotiopsis (14 compounds), Cytospora (13 compounds), Penicillium (20 compounds), and Aspergillus (35 compounds) (Fig. 16). These fungal species have been derived from different sources, including marine, endophytes, soil, cultured, and other sources. The major number of metabolites were reported from endophytic and marine-derived fungal species (Fig. 17).

These benzophenone derivatives involved simple, prenylated, and dimeric derivatives. It was found that the mixed fermentation of fungi with other microbes such as bacteria boosted the fungal production of these metabolites. Also, modification of the culture resulted in biosynthesis of new metabolites *e.g.*, 81–87 obtained from halide salts supplemented fermentation media. Therefore, these techniques could be applied for discovering new lead metabolites.

These metabolites have been assessed for various bioactivities, the major metabolites ones were evaluated for antimicrobial and cytotoxicity. It is noteworthy that limited studies investigated the anti-inflammation, anti-mycobacterial, antialgal, Plant growth inhibitory, anti-nematode, antioxidant, phytotoxic, insecticidal, antihyperlipidemic, anti-osteoclastogenic, immune-suppressive, anticoccidial, and anti-malarial, as well as α-glucosidase, proteasome, tyrosine phosphatase, protein kinase, and sterol *O*-acyltransferase inhibitory activities of these metabolites.

**Fig. 18:**
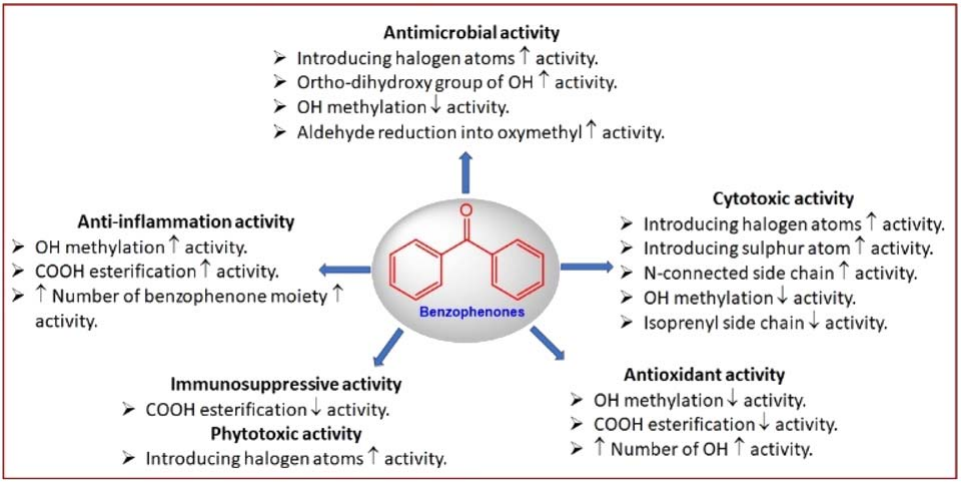
Structural features of benzophenone derivatives and structure–activity relationship (SAR) for different bioactivities.

The reported structure–activity studies revealed that the substitution pattern of these class of metabolites was greatly influenced various activities as summarized in Fig. 18.

In some of the assessed activities these metabolites revealed potent effectiveness comparable or more than that of positive control such as antimicrobial (*e.g.*, 22, 23, 37, 80, 99, 128, and 129), cytotoxic (*e.g.*, 68, 83, 84, 146, and 147), antioxidant (*e.g.*, 12, 13, 37, 38, 46, and 49), α-glucosidase inhibitors (*e.g.*, 12 and 21), anti-inflammation (*e.g.*, 72 and 137–139), antihyperlipidemic (*e.g.*, 94 and 95), and anti-osteoclastogenic (*e.g.*, 140). Further, prenylated benzophenone 69 had the ability to prohibit the cGMP dependent protein kinase activity of *E. tenella* and also revealed antiparasitic potential against TgWC (apicomplexan parasite *Toxoplasma gondii*).

It is noteworthy that limited studies exploring the mechanism of action of these metabolites were reported. For example, 140 had anabolic and/or anti-resorptive potential through osteoblast-specific genes up-regulation through BMP family members control and Smad signalling pathway that could prohibit and heal bone disorders. Compound 94 mediated anti-hyperlipidemic effect *via* SREBP-1 pathway downregulation. Compounds 146 and 147 possessed potent antimetastatic and antitumor through various mechanisms, suggesting their potential as promising new lead metabolite for finding out chemotherapeutic agents.

These metabolites worthy deserve further investigation as potential leads of therapeutic agents. The benzophenone dimerization *via* a *S*-ether functionality was greatly affected the activity, therefore, this could be a beneficial approach of synthetic research to modulate the selectivity and bioactivity of these metabolites. Future studies on the structure–activity relations, molecular mechanisms, and *in vivo* investigations of these metabolites are highly recommended.

Lastly, fungal benzophenones have diverse and often powerful bioactivities, and it is probable that more metabolites belonging to this class will be brought to light in the coming years. The creation of these metabolites through chemical synthesis could be an interesting area for future research by organic chemists.

## List of abbreviations

A549: Human lung adenocarcinoma epithelial cell line
ABTS: 2,2′-Azinobis-(3-ethylbenzthiazoline-6-sulphonate)
ACC: AcetylCoA carboxylase
AKT: Protein kinase B
BGC-823: Human gastric carcinoma cell line
BHT: Butylated hydroxytoluene
*n*-BuOH: *n*-Butanol
BT-549: Hormone-sensitive breast cancer cell line
BV-2: Microglia cells
CCD 841 CoN: Human normal colon cell line
CCD-18Co: Human normal colon cell line
CCK-8: Cell counting kit-8
CD: Circular dichroism
CH_2_Cl_2_: Dichloromethane
CHO: Chinese hamster ovary cells
Con A: Concanavalin A
CRC: Colorectal cancer cell
FAS: Fatty acid synthase
DPPH: 1,1-Diphenyl-2-picrylhydrazyl
DU145: Human prostate carcinoma cell line
EC_50_: Half maximal effective concentration
ECD: Electronic circular dichroism
EMT: Epithelial-to-mesenchymal transition
ERK: Extracellular signal-regulated kinase
EtOAc: Ethyl acetate
ESI-MS: Electrospray ionization mass spectrometry
GAPDH: Glyceraldehyde-3-phosphate dehydrogenase
GFP: Green fluorescent protein
GI_50_: The concentration for 50% of maximal inhibition of cell
H460: Human lung carcinoma cell line
H_2_O_2_: Hydrogen peroxide
HCT-116: Human colon cancer cell line
HEK293: Human embryonic kidney cell
HeLa: Human cervical epitheloid carcinoma cell line
HepG2: Human hepatocellular liver carcinoma cell line
HPLC: High-performance liquid chromatography
IC_50_: Half-maximal inhibitory concentration
IC_90_: The concentration that will inhibit 90% of the virions
IκBα: Nuclear factor of kappa light polypeptide gene enhancer in B-cells inhibitor, alpha
Kv2.1: Voltage-dependent K^+^
LD_50_: Half maximal lethal concentration
LD_90_: Lethal concentration that kills 90%
IR: Infrared
LPS: Lipopolysaccharide
Ls174T: Human colorectal cancer cell line
MCF-7: Human breast adenocarcinoma cell line
MDA-MB-231: Human breast cancer cell line
MIC: Minimum inhibitory concentrations
MNCs: Multinucleated osteoclast cells
MRSA: Methicillin-resistant *Staphylococcus aureus*
MRC5: Human lung fibroblasts
MS: Mass spectrometry
MptpB: *Mycobacterium tuberculosis* protein tyrosine phosphatase B
NFATc1: Nuclear factor of activated T cells, cytoplasmic 1
NMR: Nuclear magnetic resonance
RANKL: Receptor activator of nuclear factor-κB ligand
NO: Nitric oxide
OA: Oleic acid
ORAC: Oxygen radical absorbance capacity
p38: Multitasking kinase
PKC: Protein kinase
PANC-1: Human pancreas ductal carcinoma cell line
PC-3: Human prostatic-testosterone-independent cell line
RANKL: Receptor activator of nuclear factor kappa B ligand
RANK: Receptor activator of nuclear factor kappa B
RKO: Human colon cancer cell line
RP-18: Reversed phase-18
SF268: Human astrocytoma cell line
SRB: Sulforhodamine B
SiO_2_ CC: Silica gel column chromatography
SK-HEP-1: Human hepatic adenocarcinoma cell line
SMMC-7721: Human hepatocellular carcinoma cell line
SNU638: Human gastric cancer cell line
SOAT2: Sterol *O*-acyltransferase 2
SOAT1: Sterol *O*-acyltransferase 1
SREBP-1c: Sterol regulatory element-binding protein-1c
SW480: Human colorectal cancer cell line
TC: Triglyceride
TG: Total cholesterol
TDDFT: Time-dependent density functional theory
THP-1: Human leukemia monocytic cell line
TLC: Thin layer chromatography
U2OS: Human osteosarcoma cell line
Vero cell: Normal african green monkey kidney fibroblasts
VLC: Normal-phase vacuum liquid chromatography

## Author contributions

Conceptualization, S. R. M. I. and G. A. M.; methodology, S. R. M. I., G. A. M., S. G. A. M., and A. Y. A.; software, S. G. A. M., and A. Y. A.; writing—original draft preparation, S. R. M. I. and G. A. M.; writing—review and editing, S. G. A. M and A. Y. A. All authors have read and agreed to the published version of the manuscript.

## Conflicts of interest

There are no conflicts to declare.

## Supplementary Material
